# Inhaled Antifungal Agents for Treatment and Prophylaxis of Bronchopulmonary Invasive Mold Infections

**DOI:** 10.3390/pharmaceutics14030641

**Published:** 2022-03-14

**Authors:** Kévin Brunet, Jean-Philippe Martellosio, Frédéric Tewes, Sandrine Marchand, Blandine Rammaert

**Affiliations:** 1 Institut National de la Santé et de la Recherche Médicale, INSERM U1070, Pôle Biologie Santé, 1 rue Georges Bonnet, 86022 Poitiers, France; jean-philippe.martellosio@chu-poitiers.fr (J.-P.M.); frederic.tewes@univ-poitiers.fr (F.T.); sandrine.marchand@univ-poitiers.fr (S.M.); 2 Faculté de Médecine et Pharmacie, Université de Poitiers, 6 rue de la Milétrie, 86073 Poitiers, France; 3 Laboratoire de Mycologie-Parasitologie, Centre Hospitalier Universitaire de Poitiers, 2 rue de la Milétrie, 86021 Poitiers, France; 4 Service de Maladies Infectieuses et Tropicales, Centre Hospitalier Universitaire de Poitiers, 2 rue de la Milétrie, 86021 Poitiers, France; 5 Laboratoire de Pharmacologie-Toxicologie, Centre Hospitalier Universitaire de Poitiers, 2 rue de la Milétrie, 86021 Poitiers, France

**Keywords:** antifungal drugs, aerosol, invasive fungal disease, animal model, antifungal prophylaxis

## Abstract

Pulmonary mold infections are life-threatening diseases with high morbi-mortalities. Treatment is based on systemic antifungal agents belonging to the families of polyenes (amphotericin B) and triazoles. Despite this treatment, mortality remains high and the doses of systemic antifungals cannot be increased as they often lead to toxicity. The pulmonary aerosolization of antifungal agents can theoretically increase their concentration at the infectious site, which could improve their efficacy while limiting their systemic exposure and toxicity. However, clinical experience is poor and thus inhaled agent utilization remains unclear in term of indications, drugs, and devices. This comprehensive literature review aims to describe the pharmacokinetic behavior and the efficacy of inhaled antifungal drugs as prophylaxes and curative treatments both in animal models and humans.

## 1. Introduction

Bronchopulmonary invasive mold infections (IMI) are a major cause of mortality in immunocompromised patients such as transplant recipients and hematological patients with high-risk neutropenia [1,2]. Three main systemic antifungal classes are used to treat invasive fungal diseases. Systemic triazoles (posaconazole, itraconazole, voriconazole, isavuconazole) and polyenes (amphotericin B (AmB)) are the classes of choice for prophylaxis and treatment, but they suffer from drug-drug interactions and toxicity, while echinocandins are poorly active against molds [3].

Aerosols are an interesting route of administration, theoretically limiting systemic toxicity while ensuring high concentrations at the site of infection [4]. However, due to their physiochemical properties and pharmacokinetic characteristics, some antifungal agents are not good candidates for nebulization and should preferably be delivered intravenously. For example, AmB, which has a high molecular weight and a low permeability across biological membranes, is attractive for delivery by nebulization. Triazoles, which have a high permeability across the respiratory barrier, are less attractive to inhale as a solution, since a low residence time in the lungs is obtained. Molecules with a high respiratory barrier permeability need to be delivered as solid particles with a slow dissolution/release rate, or as advanced formulations that can control permeability to increase their residence time in the lungs after inhalation [5].

Although in some cases inhaled antifungals may be appropriate, clinical experience is still poor and leads to difficulties in their use by physicians. Indeed, the associated indications, materials used for nebulization, and suitable regimens remain unclear. In an effort to facilitate the use of inhaled antifungals, we performed a comprehensive review of their use as prophylaxes and curative treatments of pulmonary IMI in animal models and clinical cases.

## 2. Materials and Methods

Using PubMed Medline, we searched for articles in English and French that included at least one of the following groups: animal model, rat, mouse, and human. The search query used the following list of keywords: fungi, aerosol, antifungal drug, nebulized, aerosolized, aerosol, inhaled, fluconazole, flucytosine, 5-fluorocytosine, micafungin, caspofungin, echinocandin, isavuconazole, itraconazole, voriconazole, posaconazole, and amphotericin B. Articles of interest cited by the articles found were also reviewed. We included articles published from 1950. Only invasive mold infections were included in the analysis; data from the treatment of chronic or allergic bronchopulmonary aspergillosis (ABPA), fungal colonization, and severe asthma with fungal sensitization (SAFS) were excluded. Infections due to *Pneumocystis jirovecii*, yeasts, and dimorphic fungi were excluded. This search was updated on 31 December 2021.

## 3. Results

### 3.1. Selection Criteria for Choosing an Inhaler for Pulmonary Administration of Antifungals

Based on the dosing capacity of each existing technology, only dry powder inhalers (DPIs) and nebulizers are capable of delivering the high dose (usually more than 10 mg) needed to treat pulmonary fungal infections. Nebulizers can deliver several hundred milligrams of a drug to the lungs depending on the drug solubility and the tolerability of long nebulization times [6]. They can be used to deliver solutions or suspensions such as liposomal suspensions of AmB. The efficacy of nebulized antifungals to treat lung infections depends on the total amount of drug reaching the lungs and where the droplets deposit in the lungs. As IMIs are generally spread throughout the lungs, it is important to obtain sufficiently high antifungal agent concentrations all over the pulmonary tree. Liquid droplet deposition in the lungs is controlled by three main factors: airway geometry, aerodynamic particle size, and inhaled flow rate. The aerodynamic size distribution of the droplets emitted by the nebulizer is usually described by the mass median aerodynamic diameter (MMAD) and the geometric standard deviation (GSD). The MMAD refers to the diameter of the droplets above and below which 50% of the mass of drug is contained. The GSD indicates the magnitude of dispersity from the MMAD value. In general, aerosols with an MMAD between 1 and 5 µm are considered respirable [7]. These droplets are large enough to avoid elimination during exhalation and small enough to avoid deposition in the oropharynx [8,9,10]. More precisely, particles with an MMAD between 2 and 5 µm impact the upper or central airways, while particles with an MMAD of 1–2-µm deposit in the respiratory alveolar zone or peripheral airways [10,11]. Thus, the MMAD is an important parameter when choosing a nebulizer, as it allows estimation of where in the lungs the aerosol should deposit.

There are different types of nebulizers (jet nebulizers, high-frequency nebulizers often termed ultrasonic nebulizers such as vibrating mesh nebulizers, and colliding liquid jet nebulizers), which have different aerosol generation mechanisms and allow different aerosol output rates [12]. The most common nebulizers found in hospitals to treat spontaneously breathing patients are jet nebulizers, and they have been the most used in studies reporting the inhalation of antifungals [12,13]. These nebulizers use compressed air or oxygen to generate a polydispersed aerosol, with only about 5% of the aerosol being respirable, the remainder being returned to the reservoir containing the solution/suspension. In some cases, helium/oxygen (heliox) mixtures are used to reduce the resistance of the patient’s airway and, hypothetically, deliver more aerosol to obstructed airways [12]. The patient inhales the aerosol while tidal breathing from a reservoir through a mouthpiece or face mask. Hence, no specific inhalation maneuver is required. During mechanical ventilation, nebulizers can be connected to the inspiratory limb of the ventilator circuit, and the antifungal can be administered continuously or only during inspiration. In these cases, ultrasonic vibrating mesh nebulizers are attractive. They do not require compressed gas but use a high-frequency vibrating membrane filled with micrometer-sized holes to produce droplets. There is virtually no additional gas flow output from the nebulizer to interfere with ventilator operation [12].

Among the most commonly used jet nebulizers, there is a change in MMAD from 6.8 µm to 2.5 µm in standard use [14]. For example, Roth et al. tested three different jet nebulizers loaded with an AmB solution at 10 mg/mL, with droplets with an MMAD between 2 and 3 μm: Respirgard II^®^ (Marquest Inc., Englewood, CO, USA), Cirrus^®^ (Intersurgical, Wokingham, United Kingdom), and Pari IS-2^®^ (Pari-Werk, Starnberg, Germany) [15]. Jet nebulizers seem to generate particles of optimal MMAD compared to ultrasonic nebulizers. Beyer et al. demonstrated that various jet nebulizers generate AmB-loaded droplets with an MMAD of 3 to 5 μm, whereas aerosols generated by ultrasonic nebulizers contained larger droplets (MMAD > 5 μm) [8].

Besides the MMAD, other parameters such as the fine particle fraction (FPF) and the respirable delivered dose (RDD) can influence the choice of nebulizer. The FPF is the percentage of the aerosol with an MMAD between 1 and 5 μm that deposits in the lungs. For commonly used jet nebulizers, the FPF can vary from 80% to less than 40% depending on the jet nebulizer used [14]. Additionally, pulmonary doses of antifungals reaching the lungs may vary by a factor of two when comparing different nebulizers [12,16]. The RDD is calculated by multiplying the total aerosol output by the FPF, and represents the amount (mg) of respirable aerosol deposited in the lungs. When administering high doses of an aerosol, as with antifungal agents, optimizing the nebulization time may be essential to improving patient adherence to treatment. At first glance, we might consider increasing the output rate of the nebulizer to reduce the nebulization time. However, increasing the nebulizer output rate also increases the MMAD of the droplets and reduces the FPF, thereby reducing the rate of deposition of respirable aerosol [14]. For jet nebulizers, the general recommended drug solution volume is 4 to 6 mL, at a flow rate of 8 L/min [10]. In this condition, the administration lasts 10 to 20 min in clinical studies [4,17,18]. Among jet nebulizers, the breath-enhanced nebulizer system allows better drug delivery than the standard system [10].

Aerosol therapy with DPIs has gained attention in the last decade, mainly due to their ability to deliver large doses (up to 10 mg) of a drug quickly (in one puff), and due to the increased chemical stability of active molecules in a dry powder form compared to solutions. Therefore, most antibiotics approved for pulmonary inhalation are now available or under development in a dry powder form [19]. Because of the increased efficacy of aerosolized antibiotic delivery by DPIs compared to nebulizers, a much lower total dose (two to six times) is required with DPIs to achieve similar lung exposure [19]. DPIs are actuated and driven by the patient’s inspiratory flow and a powerful, deep inhalation by the DPI is required to disaggregate the powder formulation into respirable particles as efficiently as possible in order to ensure that the drug is delivered to the lungs. Therefore, powder engineering is mandatory to achieve effective aerosolization, and the off-label tests that have been used clinically to nebulize antifungal molecules from solutions intended for IV administration cannot be performed with antifungal powders which are not intended for inhalation. This has limited the clinical data available on the DPI of antifungal agents.

**Highlights (criteria for choosing an inhaler):**

- Dry powder inhalers (DPIs) and nebulizers are used to deliver antifungal agents;
- Solutions or suspensions of antifungal agents can be nebulized with nebulizers while DPIs are used to nebulize powder;
- Jet nebulizers seem to generate optimal particles compared to other nebulizers;
- Jet nebulizers use compressed gas to generate polydispersed aerosols which are inhaled by the patient through a mouthpiece or face mask. Nebulizers can be connected to the inspiratory limb of the ventilator circuit during mechanical ventilation;
- Mesh nebulizers are also widely used during mechanical ventilation.

### 3.2. Amphotericin B

Amphotericin B (AmB) is a broad-spectrum antifungal agent from the polyene family [20]. Several pharmaceutical forms of AmB exist: the historic deoxycholate formulation (AmBd), and various formulations using lipid compounds developed with the aim of limiting the nephrotoxicity of AmB. Three lipid formulations are approved by the United States Food and Drug Administration (FDA, Silver Spring, MD, USA) and the European Medicines Agency (EMA, Amsterdam, the Netherlands), and are commercially available in several countries: liposomal AmB (L-AmB), amphotericin B lipid complex (ABLC), and amphotericin B colloidal dispersion (ABCD). The antifungal effects of AmB in the lung are dose- and concentration-dependent [21,22], although AmB pulmonary diffusion is low when administered systemically [23]. Given its relatively high molecular weight (924 g/mol) and low-lipophilic properties represented by a LogD of −2.8 at pH 7.4 [23], AmB should have a long residence time in the lungs after nebulization. AmB is thus a good candidate for inhalation and was administered to humans by nebulization of various liquid formulations [24]. However, most of the clinical studies were performed using liquid AmB formulations designed for parenteral administration that have been repurposed for nebulization and used off-label to treat lower airway infections; additionally, the optimal drug dosing often remains undefined. New formulations of AmB have been recently developed for administration by DPI but in vivo studies have not been performed on these formulations [25,26].

The pharmacodynamics of L-AmB is poorly understood due to its complex pharmacokinetics [27,28]. Indeed, several measurements can be conducted: total AmB, protein bound drug, liposome associated drug, and freely circulating drug. Classically, total concentrations of AmB in both plasma and tissues are measured; however, only free AmB will be active. Consequently, the pharmacokinetic data obtained from L-AmB inhalation are difficult to analyze. The pharmacokinetics of AmBd is better understood due to the absence of liposomes. In this context, most authors refer to the minimal inhibitory concentration (MIC) to assess the efficacy of AmB in the alveolar compartments, although this is questionable due to the dose- and concentration-dependent behavior of AmB [29].

#### 3.2.1. Pharmacokinetics

**In Animal Models**

The pharmacokinetics of nebulized AmBd (n-AmBd) have been assessed in a non-infected rat model and compared to those of intraperitoneal (IP) injections [30]. Non-immunosuppressed rats were exposed to n-AmBd by jet nebulizer (air flow 8 L/min; drug solution 0.3 L/min). AmBd was efficiently delivered to the lungs while limiting accumulation in other organs. After n-AmBd nebulization of 1.6 mg/kg, concentrations were undetectable (<0.1 µg/g) in the serum, spleen, liver, kidney, and brain. Elimination from the lungs was progressive with a half-time elimination of 4.8 days after 3.2 mg/kg of n-AmBd. The maximal dose tolerated through nebulization by rats without toxicity was 60 mg/kg as determined by microscopic organ examination and animal behavior. n-AmBd pharmacokinetics have also been evaluated in a sheep model [31]. AmBd has been nebulized in animals using an ultrasonic nebulizer connected to an endotracheal tube. The drug concentration was measured in the bronchoalveolar fluid (BALF). However, the authors did not calculate the epithelial lining fluid (ELF) concentration. The peak concentration was achieved after 0.5 h in the BALF, and a slow decrease in the concentration was observed over 24 h. The peak concentrations were identical with two different doses: 5 mg and 30 mg, showing that the peak concentration of AmB in the bronchi was not directly proportional to the dose, while the area under the curve in BALF was superior with the higher dose. These data indicate that n-AmBd may reduce the systemic toxicity due to its very low systemic passage and high concentration in the lungs. Moreover, its long half-life allows several days of interval between administrations [8,32]. The distribution in the organs of nebulized L-AmB (n-L-AmB) was studied in male BALB/c 30 g mice [33]. After 1 h of nebulization of different formulations of L-AmB (8.56 to 20.03 mg of AmB) with a nose-only Collison nebulizer, L-AmB was not detected in serum at any point in time.

To find the best candidate formulation for nebulization, various formulations of AmB have been tested in similar models with divergent results. Several authors have shown that the distribution between different formulations is similar. The aerosol delivery of AmBd and L-AmB have been compared in vivo in rats [34]. The authors demonstrated that both formulations were well delivered in lungs with low systemic exposure. Both formulations led to physicochemical characteristics suitable for nebulization with the same delivered dose. In addition, liposomes are physically stable enough to be nebulized. In another naive rat model, four nebulized commercial formulations of AmB (AmBd, L-AmB, ABLC, and ABCD) were compared [35]. The distribution in the lungs after nebulization was identical whatever the formulation, and AmB was detectable after six weeks in lungs, but undetectable in serum. On the other hand, other studies have shown that L-AmB or ABLC were better candidates for nebulization with higher lung retention than AmBd. In a murine model comparing n-AmBd and n-L-AmB, the drug concentration in the lungs was 8.6 times higher with n-L-AmB [36]. In a rat model of invasive pulmonary aspergillosis, the concentration of nebulized ABLC (n-ABLC) was higher and more prolonged than that of n-AmBd in the lungs one day and seven days after treatment [37]. Moreover, n-L-AmB had no effect on surfactant function, while n-AmBd inhibited its activity [38,39]. Deoxycholic acid alone inhibited surfactant surface activity and perturbed lipid organization. Therefore, the authors proposed L-AmB as a better formulation for nebulization [39].

New formulations of AmB were developed to be used through nebulization. For example, AmB liposomes coated with alveolar macrophage-specific ligands led to a high lung concentration and a low plasma concentration in rats [40,41]. Another formulation of n-AmB using sodium deoxycholate sulfate was tested in rats [42]. Compared to n-AmBd, this formulation was less toxic for the organs, as assessed by histopathology, and provided the highest lung and plasma concentrations. Other authors have developed polymeric and lipid nanoparticles of AmB via a spray-drying technique using hydroxypropylmethycellulose and stearylamine with oleic acid [43]. This technique led to alveolar drug delivery for a long period (30–35 h).

**In Human**

In lung transplant recipients, IMIs are responsible for tracheobronchitis, bronchial anastomosis necrosis, pneumonia, and disseminated infections [44]. *Aspergillus* spp. are the most frequent fungi retrieved from the lungs of these patients. Antifungal drugs are used as prophylaxis after transplantation, and the regular control of transplant anastomosis is performed through bronchoscopy. It is thus easier to implement clinical studies using broncho-alveolar lavage (BAL) in this particular population. AmBd concentrations were measured in the bronchial secretion aspirate and BAL fluid (BALF) of 39 lung transplant recipients up to 24 h after one nebulization of 6 mg/d of AmBd, for a minimum of seven days [45]. The mean concentration in BALF was 11 μg/mL. The drug concentration in the ELF was calculated assuming that 1% of the recovered BALF corresponded to the volume of ELF. The concentration of AmB necessary to prevent *Aspergillus* is not known but the MIC of most of *Aspergillus* species is above 1 μg/mL. However, in the most proximal zone where bronchial anastomosis is located, this concentration is achieved only during the initial hours after nebulization. The proposed dosing regimen to better prevent *Aspergillus* infection of the bronchial anastomosis is thus 6 mg every 8 h during the early weeks post-transplantation [4].

Nebulized lipid formulations of AmB offer higher concentrations [23], a longer half-life, and a longer persistence in the BALF than n-AmBd, enhancing lung penetration [9]. L-AmB reaches different lung compartments after nebulization. The highest concentration was found in the alveolar compartment (24.5 ± 3.1% of total AmB introduced in the nebulizer), followed by the bronchial compartment (11.6 ± 1.3%) [46]. In two different studies in lung transplant patients, the administration of nebulized ABLC 1 mg/kg/day or L-AmB 25 mg/day resulted in high enough AmB concentrations in the ELF or BALF for *Aspergillus* spp. prophylaxis up to 7 to 14 days after the last inhaled dose [47,48]. In another study, technetium-labelled ABLC was administered using an AeroEclipse (Amherst, NY, USA) nebulizer in 12 lung transplant recipients. About 17 to 20% of the dose was deposited in the lungs. The evaluation of the size of the nebulized particles revealed a similar size to that of the *Aspergillus* conidia, and therefore, suggested a similar distribution in the tracheobronchial tract [16]. AmB concentrations of 0.50 ± 0.31 µg/mL were obtained in the ELF of eight lung transplant recipients in the 30 to 60 min following completion of the nebulization of 30 mg AmBd (5 mg/mL in 15–20 min). These concentrations are equal to the MIC of most *Aspergillus* species (i.e., 0.5 mg/L) [1].

Numerous studies have demonstrated no evidence of significant systemic absorption after nebulization (undetectable concentrations < 0.2 μg/mL at peak 1 h), under various dosing regimens (n-AmBd 6 mg/day to 10 mg/8 h; n-L-AmB 25 mg three times a week to 40 mg/day; n-ABLC 50 mg twice a week to 1 mg/kg/day) and various drug concentrations and nebulizers [8,45,47,48,49,50,51,52,53]. Only one study found therapeutic serum levels (2 μg/mL) in three out of five patients treated with n-AmBd at 20 mg b.i.d [54]. AmBd was detectable in serum (<1 μg/mL) after 10 mg b.i.d. of n-AmBd [52]. None of the four patients who received n-L-AmB at 20 mg b.i.d had detectable serum levels. In comparison, steady-state peak plasma concentrations of AmB are typically between 1.2 and 2.4 mg/mL following intravenous AmBd at 0.5–1 mg/kg, and between 7 and 12 mg/mL following intravenous L-AmB at 1 mg/kg [52]. Consequently, n-AmB led to high lung concentrations with no or very low systemic absorption. Lipid formulations seem more interesting due to their higher concentration in lungs and longer half-life, which is presumed to result in better adherence to treatment and less contamination of the nebulizer [17]. It is feasible to administer nebulized lipid formulations of AmB every one or two weeks rather than once daily for AmBd, which is more convenient for the patient. However, due to insufficient data concerning AmB concentrations at the bronchial anastomosis site in lung transplant recipients, it has been suggested that a higher frequency of administration is maintained until the suture is healed [48]. Lipid formulations are usually easier to administer as they are already in solution and do not foam, in contrast to n-AmBd [15]. Moreover, it has been shown during in vitro studies that AmBd inhibits bovine surfactant functions [38,39], whereas Monforte et al. revealed that n-L-AmB induced no changes in the lipid content of human pulmonary surfactant [55]. Albeit lower than with other systemic drugs, the cost for prophylaxis with n-L-AmB is about six fold that for a six-month n-AmBd treatment [17]. The main drawbacks of n-AmB are its unreliable distribution in the native lung in single-lung transplant recipients, who remain at risk for IMIs, due to a predominant distribution in the transplant [45], and the fact that it does not prevent extra-pulmonary infections such as candidemia.

**Highlights (nebulized AmB pharmacokinetics):**

- Nebulized AmB is well delivered in the bronchial and alveolar compartments with concentrations in the ELF or BALF above most fungal MICs;
- There is no or very weak systemic absorption;
- Lipid formulations have a higher concentration and a longer half-life in the BALF and do not alter surfactant functions; however, they are more expensive.

#### 3.2.2. Efficacy of n-AmB for Curative Treatment of IMI

**Animal Models**

To evaluate the efficacy of n-AmB, invasive pulmonary aspergillosis (IPA) models are the most studied. n-AmBd was tested on male Sprague Dawley rats (125–150 g) [56]. Rats were immunosuppressed with 100 mg of cortisone (three times per week, subcutaneously) and intratracheally inoculated with 10^6^ spores of *A. fumigatus*. One nebulization (air chamber with air flow 8 L/min; drug solution 0.3 L/min; exposure time 15 min/4.5 mL AmB; dose 1.6 mg/kg) two days after infection delayed death but did not improve survival. Comparable results were observed with one nebulization started 24 h after inoculation and continued daily for six days [56]. In the same model, combinations of one dose of n-AmBd at 1.6 mg/kg two days before inoculation with itraconazole (ITZ), SCH39304, or placebo were studied [57]. n-AmBd combined with ITZ or SCH39304 significantly improved survival at day 24 compared to n-AmBd combined with placebo.

In another study, the four commercially available formulations of AmB (AmBd, L-AmB, ABLC, and ABCD) were nebulized and compared in naive and infected rat aspergillosis model [35]. All AmB formulations were effective in prolonging survival when treatment was started 16 h after intratracheal fungal inoculation. Two studies have compared n-L-AmB and n-AmBd for IPA treatment in rats [39,58]. The two formulations prolonged rat survival. Both forms are more effective than systemic treatment, but n-L-AmB was more effective than n-AmBd to increase survival [58].

Combining n-AmB and systemic antifungal drugs could optimize IPA treatment. Results from rat aspergillosis models are, however, contradictory. One study have shown that n-L-AmB combined with systemic L-AmB did not improve survival but prevented dissemination [58]. Others have shown that n-L-AmB combined with systemic AmB (AmBd or L-AmB) is superior to either treatment alone [59]. Finally, n-L-AmB was tested in combination with intraperitoneal micafungin [60]. In total, 10 mg of n-L-AmB alone was effective to improve survival and decrease fungal burden. A combination with intraperitoneal micafungin at 1 mg/kg/day was more effective. Other formulations used for nebulization such as non-ionic surfactant vesicles containing AmB [61] or AmB polymethacrylic acid nanoparticle [62] showed good results during in vivo models. These formulations were also effective in prophylaxis.

**Clinical Studies**

Twenty-two publications, mostly case reports and case series, gathering 100 patients have reported the use of n-AmB as an adjunctive treatment for IMIs (see Supplementary Materials, Table S1) [18,63,64,65,66,67,68,69,70,71,72,73,74,75,76,77,78,79,80,81,82,83,84]. Curative nebulized treatment has been used in two forms of aspergillosis, tracheobronchial (TBA) and invasive pulmonary aspergillosis (IPA), mainly in lung cancer patients or lung transplant recipients.

n-AmBd has been used in 34 patients (Supplementary Metarials, Table S1). Different dosing regimens have been used, mostly 12.5 mg once or twice daily in recent studies, in combination with systemic anti-mold drugs and/or interventional bronchoscopic treatment and/or topical instillation of AmB [64,65,66,67,68,69,70,71]. Cure rates ranged between 36% and 67% in the two largest series [66,67]. The position of nebulization as a first line or salvage therapy was not described. Three previously described case reports involved pulmonary mucormycosis [72,73,74,75,79]. n-AmBd dosage varied from 6 mg t.i.d to 30 mg twice a week in combination with systemic AmB +/− topical instillation of AmBd +/− interventional bronchoscopic treatment or surgical treatment. All three patients were eventually cured.

Nebulized lipid formulations have been reported in a total of 67 patients (Supplementary Table S1). Peghin et al. reported a series of 22 lung transplant recipients with invasive aspergillosis (15 IPA, 7 ulcerative TBA) treated with n-L-AmB and a systemic mold-active drug. The global cure rate was 55% [18]. Safdar and Rodriguez reported a series of 32 immunosuppressed patients with various IMIs treated with n-ABLC 50 mg once or twice daily combined with various systemic antifungal agents. The global cure rate was 50% [79]. Various pulmonary IMIs including infections due to *Mucorales*, *Scedosporium* spp., *Hormographiella aspergillata*, *Fusarium* spp. and *Microascus* have been successfully treated using n-ABLC (25 to 100 mg once daily) [75,80,82] or n-L-AmB 25 mg three times a week [63,81], in combination with systemic antifungal agents and/or interventional bronchoscopic treatment and/or topical instillation of AmB. A unique publication reported the use of successful monotherapy with n-ABLC 50 mg/d in a case of IPA [77].

**Curative Treatment Guidelines**

The Infectious Disease Society of America (IDSA), the Infectious Disease Community of Practice (IDCOP), and The International Society for Heart and Lung Transplantation (ISHLT) have each issued recommendations for the use of adjunctive n-AmB in the curative treatment of IMIs [85,86,87] (Table 1).

**Highlights (nebulized AmB in curative treatment):**

- Adjunctive therapy with n-AmB has been used for pulmonary IMIs in combination with systemic drugs, but its efficacy as a primary and/or salvage therapy has not been elucidated in randomized studies;
- n-AmBd and n-L-AmB have been not compared, but animal models suggest that n-L-AmB is more efficient than n-AmBd;
- n-AmB is only recommended in association with systemic antifungal agents in TBA in lung transplants associated with anastomotic endobronchial ischemia or ischemic reperfusion injury due to airway ischemia with low evidence.

#### 3.2.3. Efficacy of n-AmB for Prophylaxis of IMI

**Animal Models**

n-AmB prophylaxis have been studied in animal models of aspergillosis, on rats [35,37,56,88,89], mice [36], and guinea pigs [90]. The only study assessing n-AmB for mucormycosis prophylaxis showed negative results with n-L-AmB [91].

n-L-AmB (0.8 mg/kg) administered two hours before challenge led to 100% survival at day eight in an aspergillosis rat model [88]. In another rat model (10^5^ *Aspergillus* spores intratracheally, discontinued corticoid immunosuppression), prophylaxis with n-AmBd administered at 1.6 mg/kg two days before inoculation decreased mortality to 11% compared to 93.8% with placebo at three weeks [89]. In the rat model previously described in the curative section [56], nebulization administered two days before inoculation delayed mortality and dramatically reduced *Aspergillus* colony forming units in the lungs.

Some authors have used new formulations of AmB such as inhalation of AmB dry powder with success in a guinea pig model of aspergillosis [90]. A single inhaled dose of dry powder at 0.05, 0.5, 4, or 10 mg/kg was administered 24 h prior to infection. This treatment improved survival and decreased the fungal burden. n-AmBd and n-L-AmB seem to be as effective regardless of the time of administration (1, 2, or 3 days) before inoculation when compared to corticoid immunosuppressed mice intranasally inoculated with *Aspergillus* spores [36]. Comparisons of the four commercialized formulations give contradictory results. They have been equally effective for survival when administered one week before inoculation in an aspergillosis rat model [35]. When prophylaxis was started six weeks before challenge, only n-L-AmB was effective, probably due to the longer half-life of L-AmB compared to other formulations. In another model, n-ABLC was more effective than n-AmBd to prolong survival [37]. A meta-analysis of n-AmB as a prophylactic for IPA on immunosuppressed animal was performed in 2015 [92]. A meta-analysis concluded that n-AmB was effective for prophylaxis in IPA with no significant variation between lipid formulations of AmB and AmBd. Moreover, they found no more adverse events (AEs) in the n-AmBd group.

**Clinical Studies in Hematology**

Most of the available studies are related to primary prophylaxis. Data on n-AmB as secondary prophylaxis for IPA in immunosuppressed patients are scarce [78].

Non-randomized studies have shown a decrease in IPA incidence rates with n-AmBd prophylaxis in high-risk hematological patients, i.e., acute myeloid leukemia (AML) and myelodysplastic syndrome (SMD) undergoing intensive chemotherapy, or autologous and allogenic stem cell transplantation (SCT) [50,93,94,95,96] (Supplementary Table S2). However, these studies lacked a control group or statistical comparison, or failed to reach significance. A prospective study of 102 patients undergoing allogenic SCT demonstrated significantly less possible/probable/proven IMI when patients received adequate n-AmBd prophylaxis (15 mg b.i.d., ≥7 days) compared with inadequate n-AmBd prophylaxis (<7 days) (2.4% vs. 18.8% at day 120, *p* < 0.05) [97]. In a retrospective cohort of 354 allogenic SCT patients receiving n-AmBd 25 mg/d as prophylaxis, the five-year incidence rate of probable or proven IA was 2.5% [98]. This is significantly lower than the 6.6% of a historical control cohort without antifungal prophylaxis. In comparison, the rate of proven and probable invasive fungal disease in AML/SMD patients undergoing induction chemotherapy was reduced from 8% to 2%, and the rate of IPA from 7% to 1% with posaconazole prophylaxis in a randomized controlled trial [99].

The only two randomized trials assessing the efficacy of n-AmBd (10 mg b.i.d.) compared to a control group in high-risk neutropenic patients did not show significant differences in the possible/probable/proven IPA incidence [100,101].

Three comparative studies assessed the efficacy of nebulized lipid formulations of AmB for prophylaxis in high-risk hematological patients (Supplementary Table S3). Two prospective studies compared prophylaxis with n-L-AmB 12.5 mg twice a week + fluconazole 400 mg/day to a historical control group treated with fluconazole 400 mg/day only [102,103]. Hullard-Pulstinger et al. found a non-significant decrease in probable/proven IA in the n-L-AmB group compared to a control group (2.1% vs. 3.8%), but few events occurred [103]. Chong et al. showed a significant decrease in probable/proven IA in the n-L-AmB group compared to the historical control group (9.5% vs. 23.4%, *p* = 0.006) [102].

One randomized double-blind controlled study evaluated the incidence rate of probable/proven IPA in 271 high-risk neutropenic patients: 139 patients received prophylactic n-L-AmB 12.5 mg twice weekly + fluconazole until neutrophil recovery > 300/mm^3^, 132 patients received nebulized placebo + fluconazole. This trial showed a significant decrease in probable/proven IPA in the n-L-AmB group (4.3% vs. 13.6%, OR 0.26, 95% CI [0.09–0.72]) [2]. A meta-analysis from 2015 showed a lower incidence of IPA among patients who underwent n-AmBd or n-L-AmB prophylaxis (OR 0.42, 95% CI [0.22–0.79], *p* = 0.007) [92].

**Clinical Studies in Lung Transplant Recipients**

Due to the lack of randomized studies, surveys of antifungal prophylaxis strategy in lung transplant centers worldwide and in the US have been published between 2006 and 2015 [104,105,106,107,108]. These studies showed great variations between centers, with an increasing use of n-AmB. In each of these surveys, most centers used universal antifungal prophylaxis. n-AmB was the most used antifungal agent, alone or in combination with oral voriconazole or itraconazole. Prophylaxis is most often initiated during the 24 h post-transplantation [104] and the duration varies from three to six months to life-long.

Non-comparative studies have shown a low incidence rate of IA < 5% with either n-AmBd or nebulized lipid formulations of AmB alone, or in association with systemic antifungal prophylaxis [18,53,109,110,111,112,113,114,115,116] (Supplementary Tables S4 and S5). The use of n-AmBd resulted in a significant decrease in IA incidence in several prospective and retrospective studies, in comparison with historical controls without antifungal prophylaxis [4,117,118,119,120].

Two studies comparing n-AmBd and n-L-AmB showed no significant differences in the incidence rates of IA [17,52] (Supplementary Table S6). A randomized double-blind trial compared the incidence rate of IFI during the first two months post-transplantation in 100 patients receiving prophylactic n-AmBd 25 mg or n-ABLC 50 mg for four days then weekly for seven weeks. Doses were doubled in mechanically ventilated patients. There was no significant difference in the incidence of IMI between the two groups, 14.3% vs. 11.8%, respectively [121]. In conclusion, the three comparative studies showed no significant differences in terms of efficacy between n-L-AmB and n-AmBd for IFI prophylaxis, even though IMI incidence rate was lower with n-L-AmB prophylaxis in all three studies [17,52,121].

**Mechanically Ventilated COVID-19 Patients**

Since patients hospitalized in critical care are at high risk for IPA, Van Ackerbroeck et al. have tested antifungal prophylaxis with n-AmBL [122]. Authors have shown that inhaled liposomal amphotericin-B (under a twice-weekly prophylactic regimen of 12.5 mg) reduced the incidence of COVID-19-associated pulmonary aspergillosis in mechanically ventilated COVID-19 patients (RR 0.15, 95% CI [0.05–0.48]).

**Prophylaxis Guidelines**

Several groups (European Society of Clinical Microbiology and Infectious Disease (ESCMID), IDCOP, ISHLT, and IDSA) have proposed guidelines for indications, dosing regimens, and the duration of n-AmB antifungal prophylaxis in hematological patients (Table 2) or transplant recipients (Table 3).

**Highlights (nebulized AmB in prophylaxis):**

- Several authors have shown the high efficacy of n-AmBd or n-L-AmBL alone or in combination with systemic drugs to prevent IPA in lung transplant recipients;
- In hematological patients with high-risk neutropenia, n-L-AmB has been associated with a decrease in probable/proven IPA incidence while the results with n-AmBd were discordant;
- In lung transplant recipients, n-AmBd or nebulized lipid formulations of AmB could be used in first or second intention;
- In hematological patients with high-risk neutropenia, n-L-AmB is recommended in second intention after posaconazole.

#### 3.2.4. Tolerance

No systemic adverse events (AEs) have been observed, especially no change in creatinine levels with either n-AmBd or n-L-AmB. Some mild and transient AEs are common with both formulations (cough, dysgeusia, nausea) with great rate differences between studies (see Supplementary Tables S2–S5), since they are dose-dependent [51]. n-AmBd AEs range from 0% to 100% of patients [4,17,49,50,51,52,54,94,95,96,97,98,100,112,119,121,126,127]. Several studies have reported an incidence of 30–40% [4,17,52,119,121] or even 50–100% of AEs [51,97,100,127]. AEs are less frequent with nebulized lipid formulations, ranging from 0% to 36.5% of patients [2,17,18,52,53,102,103,109,112,121,128] in all studies but one [129] (i.e., coughing in 74% of patients), with no severe AE. Bronchospasm and wheezing have also been reported with n-AmBd, n-L-AmB, and n-ABLC [4,17,18,51,52,54,97,121]. These AEs are rare and easily managed with salbutamol inhalation before the procedure or by halving the drug concentration [18,98]. A decline in pulmonary function has been reported in about 5% of patients (between 0 and 32%) in most studies with nebulized lipid formulations of AmB [48,53,121,128,129] and in 10.6 to 50.0% with n-AmBd [121,126]. This reversible AE has been reported both in neutropenic and lung transplant patients. Thus, local and irritative AEs of n-AmB clearly outweigh the systemic AEs observed when AmB is administered intravenously.

Treatment-limiting AEs range from 0 to 33% with n-AmBd, [17,51,97,98,100,126], and are close to 0% when using salbutamol premedication [98]. They most often range between 0 and 6% with nebulized lipid formulations [17,18,52,53,109,121,128], except in two studies that reported discontinuation rates of 42.7% [103] and 45% [2], mainly due to discomfort in the first study and patients’ weakness and technical problem in the second.

Three studies have compared n-AmBd and nebulized lipid formulations of AmB for prophylaxis in lung transplant patients. n-AmBd is associated with more AEs than lipid formulations and seems to result in more treatment discontinuations. Two non-randomized studies have shown no significant differences in the incidence of AEs and treatment-limiting AEs between n-AmBd and n-L-AmB [17,52] (see Supplementary Table S6). There is only one randomized trial comparing the tolerance of n-AmBd and lipid formulations [121]. In this study, 100 lung transplant patients were randomized to receive antifungal prophylaxis with either n-AmBd or n-ABLC for seven weeks. There were more patients who experienced at least one AE in the AmBd group than in the ABLC group (42% vs. 28%): cough (10.6% vs. 2.1%), dyspnea (19.9% vs. 2.1%), nausea (8.5% vs. 2.1%), wheezing (6.4% vs. 4.2%), dysgeusia (10.6% vs. 7.7%), and bronchospasm (25% vs. 20.4%). The decline in pulmonary function (decline of FEV1 > 20%) was similar in the two groups (10.6% vs. 11.1%). Patients who received n-AmBd were more likely to experience AEs (OR = 2.16, 95% CI [1.10–4.24], *p* = 0.02). Treatment-limiting AEs were higher in the AmBd group (12.2% vs. 5.9%), although this finding did not reach significance due to a lack of power [121]. A meta-analysis of these three prospective studies in lung-transplant recipients was unable to compare the AEs of n-AmBd and nebulized lipid formulations due to non-uniform reporting of the data [130]. In terms of treatment-limiting AEs, meta-analysis showed a 4% rate with nebulized lipid formulations vs. 8% for n-AmBd not reaching significance (HR 0.57, 95% CI [0.22–1.50]).

**Highlights (nebulized AmB tolerance):**

- Global tolerance of n-AmB is good with no or very few systemic AEs;
- Mild and transient AEs are common with both formulations (cough, dysgeusia, nausea);
- Bronchospasm and wheezing are rare and easily managed with salbutamol inhalation or by halving drug concentration;
- Decline in pulmonary function is rare but has been reported in about 5% of patients;
- AEs are less frequent with lipid formulations.

### 3.3. Triazoles

#### 3.3.1. Voriconazole

Voriconazole (VRZ) is a relatively lipophilic molecule with a LogD = 1.8 at pH 7.4, with an ELF/plasma ratio between 6 and 11 when administered by a systemic route in humans [23]. Several studies have investigated the aerodynamic properties of nebulized voriconazole (n-VRZ) using commercially available solutions concentrated at 6.25 to 10 mg/mL. The MMAD was 2.4 to 2.98 μm and the FPF was 71.7–93.0% [131,132,133,134]. These results suggested an appropriate distribution of the nebulized droplets into the distal zones of the lung but a rapid systemic absorption.

The pharmacokinetics of n-VRZ was investigated in non-infected racing pigeons [135]. After nebulization for 15 min at 10 mg/mL of an intravenous commercially available solution, the authors showed a low concentration in the lungs with no detection after one hour and a fast decrease in plasma. A non-commercial aqueous solution of VRZ solubilized with sulfobutyl ether-beta-cyclodextrin was nebulized in mice [136]. High concentrations were observed in the lungs and plasma within 30 min. The ratio of lung/plasma concentration was 1.4 after a single inhaled dose and 2.9 after multiple doses. A rapid distribution from lung to blood was observed. In another model, chronically inhaled voriconazole was well tolerated in a rat model [133].

A rapid distribution of n-VRZ from lungs to plasma was also observed in humans. Therapeutic drug levels in human lung tissues occurred 30 min after inhalation of a solution at 10 mg/mL, and maximal pulmonary concentrations were 1.4 times higher than plasma concentrations [133,134]. In a study including six patients, n-VRZ was rapidly absorbed into systemic circulation with detectable serum concentrations 15 min after nebulization of 40 mg. Median plasma concentrations were 100 times lower twelve hours after the last nebulization in the inhalation group, who received 40 mg b.i.d for two days, compared to the oral group (400 mg b.i.d at day 1, 200 mg b.i.d at day 2) (8 vs. 1224 ng/mL, *p* < 0.0001) [137]. There was a non-significant trend towards a higher median ELF/plasma concentration ratio in the inhalation group (21, 95% CI [6,7,8,9,10,11,12,13,14,15,16,17,18,19,20,21,22,23,24,25,26,27,28,29,30,31,32,33,34,35,36,37,38,39,40,41,42,43,44,45,46,47,48,49,50,51,52,53,54,55,56,57,58,59,60,61,62,63]) compared to the oral group (8, 95% CI [3,4,5,6,7,8,9,10,11,12,13,14,15,16,17,18,19,20]; *p* = 0.2).

Different formulations have been tested to try to retain VRZ in the lungs, such as nanoparticles [138], dry powder insufflation of crystalline and amorphous VRZ formulations [139], polylactic-co-glycolic acid (PGLA) nanoparticles [140], chitosan-coated PGLA nanoparticles [141], and inhalable dry powder by spray freeze drying [142]. These formulations showed good aerosol properties with efficient lung deposition and higher concentrations in the lungs than intravenous VRZ. These formulations led to clinically relevant concentrations in plasma but with variable systemic absorption.

n-VRZ has solely been studied for curative treatment in humans and not for prophylaxis. One mouse model of IPA showed that aqueous solutions of VRZ solubilized with sulfobutyl ether-beta-cyclodextrin improved survival when administered as prophylaxis and were more efficient than IP injection of AmB [134]. In humans, four case reports have been reported thus far using n-VRZ as an adjunctive treatment of IMI (see Supplementary Table S7). Two publications involving three lung transplant recipients and one immunosuppressed patient with IPA showed clinical and/or radiological cure or improvement with n-VRZ 40 mg administered one to three times daily, alone or as adjunctive treatment [131,132]. n-VRZ 40 mg once or twice daily has also been used as an adjunctive treatment for *Scedosporium apiospermum* and *Microascus* sp. invasive infections with good results [82,143].

**Highlights (voriconazole):**

- Nebulized voriconazole is well delivered in lungs but leads to rapid systemic absorption;
- The ELF/plasma concentration ratio is not significantly different between nebulized and oral voriconazole.

#### 3.3.2. Itraconazole

Itraconazole (ITZ) is a highly lipophilic molecule with a ratio of 3:1 in lung tissue versus plasma when administered systemically [144]. Due to this lipophilic characteristic, it must be solubilized to be aerosolized [145].

Nanoparticles of ITZ were synthetized with different technologies: evaporative precipitation of aqueous solution (EPAS), spray freezing into liquid (SFL), and ultra-rapid freezing (URF) [146]. These formulations allow ITZ to rapidly dissolve. Lung deposition was similar between EPAS and SFL, with high lung concentrations [147]. The amorphous nanoparticles produced by SFL showed higher concentrations in the lungs and serum than oral solutions [148]. The aerosolization of amorphous ITZ produced by SFL has no pro-inflammatory effect nor tissue toxicity [149]. A nanostructured ITZ solid solution was developed by URF technology [150]. This formulation led to high lung deposition but high systemic concentration. The efficacy of these formulations was assessed in experimental models. The EPAS and SFL of ITZ both achieved high lung concentrations with reduced systemic exposure in a murine model of IPA [151]. These forms of ITZ were more effective in prophylaxis for *A. flavus* aspergillosis than oral suspension or placebo. SFL showed better results than EPAS in survival studies. Aerosolized nanostructured formulations of ITZ produced by SFL were tested in aspergillosis prophylaxis models and compared with ITZ by oral gavage [152]. The aerosolized formulation was used two days before inoculation and continued for seven days post-inoculation. This treatment was more effective than ITZ by oral gavage for improved survival, and histopathological examination of lung biopsies displayed less lesions. The pulmonary delivery of an ITZ cyclodextrin solubilized solution was compared with colloidal dispersion of an ITZ nanoparticle formulation synthetized by URF [153]. These two formulations had the same lung deposition with deep lung delivery and the same pharmacokinetic profile. ITZ cyclodextrin solubilized solution had, however, a more rapid systemic distribution [153]. The authors also compared the bioavailability of amorphous ITZ nanoparticles synthetized by URF versus crystalline ITZ nanoparticles produced by wet milling [154]. Although both formulations allowed similar deep lung delivery due to compatible aerodynamic diameters, amorphous nanoparticles of ITZ showed a higher systemic bioavailability.

Using a wet-milling process with organic milling beads, a stable nanosuspension of ITZ at 20% was synthetized [155]. Nebulization of this suspension led to a high and long-lasting lung concentration with a minimal systemic exposure in rats. A single dose of 22.5 mg/kg led to 25.4 h of half-life. These nanosuspensions were tested in Japanese quail [156]. This formulation permitted high lung concentrations with a low systemic exposure in young quail. Moreover, a nanosuspension of ITZ at 10% or 4% was effective for aspergillosis treatment in this model [157]. Treatment was once daily for 30 min, starting 2 h after inoculation for six days, which increased survival and was well tolerated.

More recently, some authors synthetized ITZ-loaded nanostructured lipid carriers for pulmonary treatment of IPA in falcons [158]. This formulation can be easily nebulized and effectively penetrated the respiratory tract.

Several authors have evaluated the endotracheal insufflator device for the administration of dry powder and have shown that it can be used in preclinical trials [159]. This material was used to test three ITZ dry powders for inhalation [160]. ITZ was prepared by spray-drying a mannitol solution in which ITZ was dispersed or solubilized. The concentrations of ITZ in lungs were high for two of the formulations but with high systemic bioavailabilities. Micronized cocrystal powders, micronized using the jet-milling system with succinic acid (SA) or l-tartaric acid (TA), and amorphous spray-dried formulations of ITZ were also evaluated [161]. Micronized cocrystals are promising formulations for enhancing the pulmonary absorption of poorly soluble compounds.

Finally, the relationship between the in vitro dissolution of ITZ powder and its fate in vivo was assessed [162]. The authors showed that the dissolution of ITZ in the lungs may be increased to avoid local irritation and rapid elimination by macrophages. However, high dissolution led to a fast systemic absorption and a lower lung retention.

**Highlights (itraconazole):**

- Nebulized itraconazole is well delivered in lungs but leads to rapid systemic absorption;
- Advanced formulations are in development to control the permeability and/or the release rate in pulmonary compartments.

#### 3.3.3. Other Triazoles

The aerodynamic properties of nebulized molecules of IV posaconazole solutions have been characterized. With an MMAD of 3.0 to 3.4 µm and a FPF of 78–79% for solutions concentrated at 6 to 12 mg/mL, an appropriate distribution in the lungs is suggested [132]. Alhough posaconazole is a lipophilic molecule with a LogD of 2.15 at pH 7.4, it is not a good candidate for nebulization. Nebulized posaconazole has not been tested to treat IMIs.

A new triazole drug, named PC945 (opelconazole), was developed to optimize topical treatment with tissue retention and physicochemical properties adapted to inhalation [163,164]. Opelconazole inhalation resulted in high local concentrations, prolonged lung retention, slow absorption from the lung, and low plasma concentrations [165]. Intranasal PC945 showed an increased survival in a murine model of IPA. This compound was able to decrease the fungal load and in vivo biomarkers of aspergillosis in the BAL and serum and showed superiority to voriconazole and posaconazole [166]. Moreover, the drug acted synergistically with commonly used triazoles (posaconazole, VRZ, ITZ) [167]. Phase I and IIa were recently performed and showed only mild-to-moderate AEs in 29 subjects. Opelconazole was used in a compassionate program on nine patients and positive clinical results were observed in eight [164]. Since opelconazole was poorly absorbed, the drug should not be used alone in disseminated forms of IMI but could represent a new option of great value in combination with systemic treatment or in prophylaxis.

Another new triazole drug, PC1244, showed efficacy against IPA when administered intranasally [168]. This compound showed better activity than VRZ and posaconazole against azole-resistant *Aspergillus* [169].

**Highlights (other triazoles):**

- As with other triazoles, posaconazole nebulization leads to rapid systemic absorption;
- New triazole antifungal agents showed efficacy in nebulization in IPA animal models.

### 3.4. Other Antifungal Agents

Among other antifungal agents, imidazoles (ketoconazole, miconazole, prochloraz) are not used as aerosolized agents since they lead to histamine release and airway constriction [170].

Since echinocandins are poorly effective against molds, few studies have been performed on this topic. However, pharmacokinetic data have shown that caspofungin is well delivered to the lungs with higher maximal concentrations and AUC compared to IV routes [171]. Several authors have developed new formulations of caspofungin to improve alveolar concentrations [172]. Inhaled micafungin was used for the treatment of two *Scopulariospsis/Microascus* infections and led to high ELF concentrations with low plasmatic passage and was well tolerated [173].

Pneumocandin L-693,989, an experimental echinocandin, administered at 5 mg/kg 2 h before inoculation was effective in improving survival in a rat model of IPA [88]. In another rat model of IPA, an aerosol of hamycin at 0.68 mg/kg administered two days before infection delayed mortality compared to the control group [174]. Administered as a curative treatment, at the same dosage of 0.68 mg/kg 24 h after inoculation daily for six days, nebulized hamycin was effective.

The pharmacokinetics of nebulized terbinafine was assessed in Hispaniolan Amazon parrots. The time above MIC remained low in the study conditions [175]. The lack of efficacy of this drug as prophylaxis was demonstrated in an aspergillosis rat model nebulized with terbinafine at 1.6 mg/kg two days before inoculation [176].

New drugs, some belonging to new families, are actually under development. These new drugs include fosmanogepix (a novel Gwt1 enzyme inhibitor), ibrexafungerp (a first-in-class triterpenoid), and olorofim (a novel dihydroorotate dehydrogenase enzyme inhibitor) [164]. The use of these drugs by nebulized routes should be evaluated to bring new families of inhaled drugs.

### 3.5. Perspectives/Recommendations for Future Research

- Perform prospective randomized studies to evidence the efficacy of n-AmB in the prophylaxis and curative treatment of IMIs, especially in particular settings such as severe or refractory infections, or large cavitary lesions;
- Perform prospective randomized studies to compare the efficacy and tolerance of lipid formulations of AmB and AmBd;
- Continue the development of opelconazole;
- Test new antifungal agents (fosmanogepix, ibrexafungerp, olorofim) by nebulized route;
- Evaluate n-AmB on emerging IMIs such as mucormycosis.

## 4. Conclusions

Animal models and clinical data have shown that AmB presents adequate physicochemical characteristics for nebulization compared to other antifungal drugs which need appropriate formulations to be retained in the lungs. n-AmB leads to high alveolar concentrations and no or very low systemic passage. Most animal studies have proposed lipid forms of AmB as the best candidates for nebulization due to better lung penetration, a higher half-life, and the improvement of survival. The choice of nebulizer is crucial in humans; jet nebulizers seem to generate more optimal particles compared to ultrasonic nebulizers. Combining systemic treatments for nebulization could prevent fungal dissemination. n-AmB was thus tested as an adjunctive therapy, as a primary and salvage therapy for refractory pulmonary IMIs in patients. Although the results seem to favor increased efficacy, no randomized controlled prospective studies have elucidated the impact of n-AmB on survival. In prophylaxis, several authors showed a high efficacy of n-AmB to prevent IA. In hematological patients with high-risk neutropenia, even if data are scarce, n-L-AmB showed a decrease in probable/proven IPA incidence. Global tolerance of n-AmB is good, with no or very few systemic AEs, bronchospasm, and wheezing being less frequent with lipid formulations.

Prospective randomized studies are mandatory to evidence the efficacy of n-AmB in prophylaxis and curative treatments. To recommend n-AmB outside of salvage therapy or prophylaxis in lung transplant recipients is complex due to the lack of evidence. Moreover, most data come from IPA. It would be interesting to evaluate the effect of n-AmB on emerging IMIs such as mucormycosis. Prospective randomized studies would also be useful to compare the efficacy and tolerance of lipid formulations of AmB and AmBd. Finally, the development of opelconazole must be continued. Indeed, it could represent a new option in the treatment of IMIs by nebulized routes.

## Figures and Tables

**Table 1:** Guidelines for the use of nebulized AmB in treatment for invasive mold infections in lung transplant recipients.

Ref	Type of IFD	n-AmB	Evidence
Patterson, 2016 [86]	TBA in lung transplants associated with anastomotic endobronchial ischemia or ischemic reperfusion injury due to airway ischemia	Adjunctive inhaled AmB is recommended in association with a systemic antimold antifungal (strong recommendation; moderate-quality evidence).	“No consistent evidence”
Husain, 2016 [87]	TBA	n-AmB alone is not recommended as a primary treatment of TBA (C-III). Although it has been proposed as an adjunctive therapy in an endobronchial prothesis infection, more evidence is needed.	Morales, 2009 [80]
	IPA	Addition of n-AmB to a standard regimen of treatment is not routinely recommended (C-III). However, the authors also declare that n-AmB could be used in combination with voriconazole/other systemic antifungal drugs, depending on the severity of IFD, or possibly in situations in which large cavitary lesions might render the penetration of systemic agents difficult.	Additional evidence would be helpful
Husain, 2019 [85]	TBA associated with anastomotic endo-bronchial ischemia, or ischemic reperfusion injury due to airway ischemia associated with lung transplant	Inhaled AmB (in conjunction with systemic antifungal therapy) may be used (weak; low).	

**Table 2:** Guidelines for prophylactic use of nebulized AmB in hematological patients.

Ref	Criteria	n-AmBd	n-L-AmB
Mellinghoff, 2018 [123]	Neutrophils < 500/mm^3^, >7 days. (AlloSCT and ALL excluded)	Recommendation against its use (D-I)	Recommended B-II (second choice after posaconazole) 12.5 mg × 2/week in combination with fluconazole 400 mg/day
Patterson, 2016 [86]	Prolonged neutropenia (induction/reinduction therapy for AL, and alloSCT recipients following conditioning or during treatment of GVHD)	n-AmB may be considered (weak recommendation; low-quality evidence) AmB lipid formulations are generally better tolerated than AmBd	
Ullman, 2018 [124]	Prolonged and profound neutropenia	Not mentioned	Recommended B-I (second choice after posaconazole) 12.5 mg × 2/week in combination with fluconazole 400 mg/day
	AlloSCT recipients until neutrophil recovery	Not mentioned	Recommended B-II (ex-aqueous first choice with posaconazole) 12.5 mg × 2/week in combination with fluconazole 400 mg/day
Maertens, 2018 [125]	Induction AML/MDS	Recommendation against its use (A-I)	Recommended B-I (second choice after posaconazole) 10 mg × 2/week in combination with fluconazole 400 mg/day
	AlloSCT with high-risk mold infection		Not recommended if low incidence of mold infections (<5%), recommended B-II (third choice) if high incidence of mold infections (>5%) 10 mg × 2/week in combination with fluconazole 400 mg/day

AlloSCT: allogeneic stem cell transplantation; ALL: acute lymphoblastic leukemia; AML: acute myeloid leukemia; MDS: myelodysplastic syndrome; AmB: amphotericin B; n-AmB: nebulized AmB; AmBd: deoxycholate AmB; L-AmB: liposomal AmB.

**Table 3:** Guidelines for prophylactic use of nebulized AmB in lung transplant recipients.

Ref	Criteria	n-AmBd	n-AmB Lipid Formulation
Husain, 2016 [87]	Lung transplant recipients	n-AmB ± fluconazole or an echinocandin should be used in the first 2–4 weeks post-transplantation (B-I)	
Patterson, 2016 [86]	Lung transplant recipients	n-AmB may be considered (weak recommendation; low-quality evidence) AmB lipid formulations are generally better tolerated than AmBd	
Ullman, 2018 [124]	Lung transplant recipients	B-II 25 mg/day for 4 days, followed by 25 mg/week for 7 weeks	Recommended A-I (first choice) More AEs with AmBd but similar efficacy; various possible protocols: ABLC 50 mg/d for 4 d, then 50 mg/w for 7 w. ABLC 50 mg/day for 2 w., then 1×/w for 10 w. L-AmB 25 mg × 3/w. (day 1–60) post SOT, then 1×/w. (day 60–180)
	Heart transplant recipients	n-AmB universal prophylaxis is recommended in second choice (C-I). First choice is targeted prophylaxis with echinocandins	
Husain and Camargo, 2019 [85]	Lung transplant recipients	AmBd 20 mg × 3/d or 25 mg/d (weak; low)	ABLC 50 mg 1×/2d for 2 w., then 1×/w for 13 w. (week; low) L-AmB 25 mg × 3/w. for 2 months, then 1×/w. for 6 m., then 2×/m. thereafter (weak; low)
	Heart transplant recipients	Not cited Targeted prophylaxis with itraconazole or voriconazole or echinocandins is recommended in patients at risk	

AmB: amphotericin B; n-AmB: nebulized AmB; AmBd: deoxycholate AmB; L-AmB: liposomal AmB; SOT: solid organ transplantation; AEs: adverse events.

## Supplementary Materials

The following are available online at https://www.mdpi.com/article/10.3390/pharmaceutics14030641/s1, Table S1: Nebulized AmB used as curative treatment for invasive mold infections, Table S2: Nebulized AmBd as prophylaxis in hematological patients, Table S3: Nebulized lipid formulations of AmB (L-AmB or ABLC) as prophylaxis in hematological patients, Table S4: Nebulized AmBd as prophylaxis in lung transplant recipients, Table S5: Nebulized AmB lipid formulations as prophylaxis in lung transplant recipients, Table S6: Comparative studies of nebulized AmBd prophylaxis and nebulized lipid formulations of AmB prophylaxis, Table S7: Studies of n-voriconazole used as curative treatment for invasive mold.
