# Supplementary material for: Inhaled Antifungal Agents for Treatment and Prophylaxis of Bronchopulmonary Invasive Mold Infections
Source: Pharmaceutics. 2022 Mar 14;14(3):641. doi: 10.3390/pharmaceutics14030641 (PMC8949245; doi:10.3390/pharmaceutics14030641)
Supplement: Supplementary file 1 [file pharmaceutics-14-00641-s001.zip › pharmaceutics-1602259-supplementary.pdf]

pharmaceutics

Supplementary materials: Inhaled Antifungal Agents for Treatment and Prophylaxis of Bronchopulmonary Invasive Mold Infections

Kévin Brunet, Jean-Philippe Martellosio, Frédéric Tewes, Sandrine Marchand and Blandine Rammaert

List of acronyms

* $p<0.05$

+: plus

ABLC: amphotericin B lipid complex

AE: adverse event

ALL: acute lymphoblastic leukemia

AmBd: amphotericin B deoxycholate

AmBLF: amphotericin B lipid formulation

AML: acute myeloid leukemia

BID: two times a day

CLL: chronic lymphocytic leukemia

COPD: chronic obstructive pulmonary disease

D: day

FEV1: forced expiratory volume in one second

FLU: fluconazole

IA: invasive aspergillosis

IFD: invasive fungal disease

IPA: invasive pulmonary aspergillosis

ISC: isavuconazole

ITR: itraconazole

L-AmB: liposomal amphotericin B

M: month

MDS: myelodysplastic syndrome

MICA: micafungin

MMAD: median mass aerodynamic diameter

N: number of patients

N-AmB: nebulized amphotericine B

NR: not reported

NS: non-significant

OID: once a day

PMN: polymorphonuclear neutrophils

PSC: posaconazole

REF: reference

SCT: stem cell transplantation

TBA: tracheobronchial aspergillosis

TID: three times a day

VORI: voriconazole

W: week

Y.: year

Table S1. Nebulized AmB used as curative treatment for invasive mold infections.

Ref	N	Comorbidity	Pathogen	Pathology	Drug and Dilution	Dose[a]	Nebulizer	Other treatments	Outcome[b]
Birsan 1998 (1)	1	NR	*Aspergillus* sp.	TBA	AmBd, dilution n.d.	10 mg t.i.d, 6-8 w.	NR	Itraconazole PO	Cured
Zhou 2017 (2)	1	42y., None except *influenzae* infection	*Aspergillus* sp.	IPA	AmBd 25mg in 15mL of water for injection	12.5mg ×2/d, 3w.	NR	Voriconazole 16 w.	Clinical and Radiological cure
Huang 2012 (3)	11	Cancer (n=10, 5 lung cancer, 3 trachea), lung transplant and COPD (n=1)	*Aspergillus* sp.	TBA (necrotizing)	AmBd 12.5mg in 3 5mL of normal saline	12.5 mg/d., 15w. to 1y.[c]	NR	Interventional bronchoscopic treatment (all) + various systemic antifungal agents and eventually topical instillation of AmB	36% cured or improved
Wu 2010 (4)	12	Cancer (n=8, 5 lung, 1 trachea), lymphoma (n=1), none (n=2), tracheostenosis (n=1)	*Aspergillus* sp.	TBA (invasive : ulcer or necrosis or pseudomembranes)	AmBd dilution n.d.	5-10 mg ×2/d, 8 to 47d.[d]	NR	Various systemic antifungal agents and eventually interventional bronchoscopic treatment and eventually topical instillation of AmB	67% cured

Dal Conte 1996 (5)	1	33y., AIDS	*Aspergillus* sp.	TBA (invasive : positive biopsies and alveolar infiltrates)	AmBd 10 then 20 mg in 10mL of sterile water	10 then 20 mg/d, 45d.	Respiguard II	Itraconazole 400mg/d, flucytosine 21d.	Clinical and radiological improvemen, negative culture, patient died 2w. after dischar e from *P.aeruginosa* pneumonia. Good tolerance.
Boots 1999 (6)	1	35y., none except *influenzae* infection	*Aspergillus* sp.	TBA (invasive: positive biopsies, necrosis, membranes)	AmBd, dilution n.d.	10mg ×2/d, 56d	NR	Various systemic antifungal agents	Cured
Rodenhuis 1984 (7)	1	48y., alcohol abuse and asthma	*Aspergillus* sp.	IPA	AmBd in 3mL of 5% dextrose	Increased from 1.25mg ×4/d to 25mg ×4/d, 8w.	Hudson 1720 Updraft	Flucytosine PO 150mg/d and AmBd 50mg/d IV, 8w.	Clinical cure and radiological improvement
Boettcher 2000 (8)	3	Lung transplant (n=3)	*Aspergillus* sp.	TBA (ulcerative)	AmBd (n=1)	10mg b.i.d	NR	IV L-AmB 150-200mg/d and IV flucytosine 2.5mg t.i.d or q.i.d + secondary adjunction of topical instillation of AmBd or L-AmB and eventually interventional bronchoscopic treatment	1 died (AmBd) from arterio-bronchic
					L-AmB (n=2)	50mg t.i.d, Duration: 12w. in case 2, n.d. in case 3			Fistula 2 cured (sequelary anastomotic stenosis)
Furco 2001 (9)	1	66y., diabetes	*Mucorales* hyphae (culture not performed)	Pulmonary mucormycosis	AmBd 30mg in 5mL of saline serum	30mg ×3/w, for 3 months	Europe Médical, O2 5L/min	IV L-AmB 150mg/d	Cured
Alfageme 2009 (10)	1	21y., induction treatment for ALL	*Mucor* sp.	Pulmonary mucormycosis	AmBd	n.d.	NR	IV L-AmB, adjunction of topical instillation of AmBd, adjunction of posaconazole, surgical treatment	Radiological improvement after topical instillations of AmBd, cure after surgical treatment
McGuire 2007 (11)	1	Bilateral lung transplant	*Rhizomucor* sp.	Mucormycosis of the bronchial anastomosis	AmBd	6mg t.i.d, indefinetely	NR	IV AmBd for 4 months + interventional bronchoscopic treatment	Clinical cure and normalized pulmonary function tests. Sequelary anastomotic stenosis dilatated.
Safdar 2004 (12)	1	61y., allogenic stem cell transplant	*Pseudallescheria boydii* and *Rhizomucor* sp.	Pulmonary IFD	ABLC	50mg/d for 5 months	Respiguard II	Caspofungine 50mg/d	Clinical and radiological-cure.

									Good tolerance.
Godet 2017 (13)	1	36y., allogenic stem cell transplant	*Hormographiella aspergillata*	Pulmonary IFD	L-AmB	25mg ×3/w	NR	L-AmB 3mg/kg/D	Clinical Cure and radiological improvement
Castagnolla 2007 (14)	1	8y., allogenic stem cell transplant	*Aspergillus sp.*	Refractory IPA	L-AmB	20mg ×2/d for 3d., then 15mg ×2/d. Stopped after 8 days because of neurological AE.	NR	L-AmB IV 10mg/kg/d + Caspofungine IV 100mg/m2	Clinical and radiological improvement after adjunction of n-L-AmB. Died 2 years later from secondary AML
Canetti 2015 (15)	1	67y., transplant kidney	*Aspergillus sp*	IPA	ABLC	50mg/d monotherapy after voriconazole suspension. Gradually reduced and suspended after 13 months	Mede jet (Medel PRO®) nebulizer	Voriconazole 200mg ×2/d stopped after 50d. due to liver toxicity	Radiological improvement, B-D- Glucan normalization. No AE.
Venanzi 2019 (16)	5	2 allo SCT, 1 AML, 2 cancer and COPD	*Aspergillus sp*	IPA	ABLC	50mg/d (5mg/ml), sequentially reduced to 50mg ×3/w, 50 mg ×2/w, 50 mg /w in patients with a favorable outcome.	PARI BOY SX nebulizers (PARI GmbH, Germany) and eFlow rapid nebulizer (PARI GmbH, Germany)	Systemic voriconazole or L- AmB or Caspofungin	3 complete response, 2 deaths not related. No AE
Safdar et Rodriguez 2013 (17)	32	Allogenic stem cell transplant (56%), acute leukemia (69%), severe neutropenia (63%)	*Aspergillus* sp. (n=7), *Fusarium* sp. (n=2), *Rhizmucor* sp., *Scedosporium* sp., *Basidiomycete* sp. (n=1 each)	Possible, probable or definite IFD	ABLC 50mg in 10mL of saline serum	50mg x2/d (78%) or 1x/d (22%) Median duration : 28 d	Small-volume jet-nebulizers yielding 80% drug with less Than 5-µm Particle size Were used, preceeded by 1 dose of SABA	Various systemic antifungal agents	Complete or partial clinical and radiographic resolution in 50%. No serious adverse effect
Morales 2009 (18)	1	33y., bilateral lung transplant with endobronchial prothesis	*Aspergillus* sp., *Scedosporium* sp.	TBA / infection of endobronchial prothesis	ABLC	25mg o.i.d	NR	Voriconazole 400mg/d + interventional bronchoscopic treatment +	Clinical and microbiological cure (only

				(with local laceration)				adjunction of topical instillation of AmBd or AmBLF	after instillations were added)
Al Yazidi 2019 (19)	1	13y., bilateral lung transplant	*Fusarium* sp.	Infection of the bronchial anastomosis (pseudomembranes)	L-AmB	25mg x3/w, indefinitely	NR	Posaconazole 300mg/d then 500mg/d	L-AmB induced ronchospasm managed with salbutamol
Peghin 2016 (20)	22	Lung transplant	*Aspergillus* sp.	IPA (n=15), ulcerative TBA (n=7)	L-AmB 50mg in 12 mL of sterile water	NR	Mainly jet nebulizer (Ventstreamor ® Sidestream®, Phillips Respironics, with a CR60 compressor (air pressure, 27.2 psi; flow, 7.3 L/min; Phillips Respironics) equipped with a disposable bacterial exhale filter	Systemic voriconazole and/or caspofungin and/or L-AmB	Successfull therapy in 55%
Taton 2018 (21)	1	60y., bilateral lung transplant	*Microascus* sp.	Bronchial IFD	Voriconazole And ABLC	40mg b.i.d and 100mg o.i.d; duration: 7w.	NR	Combination of IV voriconazole, caspofungin and terbinafine + interventional bronchoscopic treatment	Clinical and bronchoscopic cure
Trujillo 2021 (22)	1	Kidney transplant, severe COVID-19	*A. fumigatus*	IPA	n-AmBL	25 mg [6 mL] thrice weekly	NR	ISC	Evolution was favorable

[a] including treatment duration when notified in the article, [b] including toxicity data when available, [c] mean duration of treatment was 3.5 months in responsive patients, [d] mean duration of treatment was 25 days, NR: not reported.

Table S2. Nebulized AmBd as prophylaxis in hematological patients.

Ref.	Study design	Comorbidities	Dose, Nebulizer	Adverse events / severe AE / treatment–limiting AE / type of AE	Outcome
Conneally 1990 (23)	Prospective, N=34 (vs 123 historical control)	Neutropenic oncology, SCT, chemotherapy for hematological malignancies	AmBd 5mg bid	NR	0 vs 11.4% of IA over 1 year
Jeffery 1991 (24)	Retrospective, N=130.	Acute leukemia 57%, SCT 27%	AmBd 5mg/d, 1mg/ml, De Vilbiss atomizer	No AE noted	11.2% of proven IA over 11 years. 0 IFD over the 3 last

	AmBd prophylaxis (1986-1988) vs historical control (no prophylaxis:1977-1986)		(model 251 atomizer - De Vilbiss, Somerset, Pennsylvania)		years period when AmBd prophylaxis was used.
Myers 1992 (25)	Non comparative, N=26	SCT (69%), leukemia (31%)	5mg bid to 20mg bid (5mg/ml) ; Airlife" Misty-Neb Medication System	3.8% (bronchospasm) / 0 / 3.8%	No IA over a 4 months period
Gryn 1993 (26)	Non comparative, N=29	Leukemia or SCT	30mg oid (10mg/ml), Misty Neb (Baxter Healthcare)	100% / 0 / 7%<br>AEs: Coughing and dysgeusia (100%), nausea (69%), wheezing (17%), dysphagia (14%)	NR (toxicity study)
Beyer 1993 (27)	Prospective non comparative N=40	Auto-SCT or high dose chemotherapy for germ cell tumors	AmBd 10mg bid; 2mg/ml; RespirGard II nebulizer and the Lifetec Jetair 10 compressor	AEs: Dysgueusia, mild nausea, discomfort	5% of probable/proven IPA
Heinemann 1994 (28)	Prospective non comparative, N=22	AML 77%	10mg t.i.d, 5mg/ml	Treatment was well tolerated, and compliance was generally good. Apart from a slight bronchial irritation during deep inhalation, toxic pulmonary AEs were not observed. Median creatinine values unchanged.<br>No serum AmB detected (n=10)	No positive *Aspergillus* culture from weekly performed analysis (nose, throat, sputum)
Hertenstein 1994 (29)	Prospective non comparative, N=303	55% AML, 26% CML, 19% other 89% alloSCT, 9% autoSCT	10mg bid	Non limiting occasional mild cough and dysgueusia	3.6% proven IFD over a 120 days period
Behre 1995 (30)	Randomized, 65 (AmBd prophylaxis) vs 50 (no prophylaxis)	AML 58%, solid tumor hd chemotherapy (34%)	10mg bid (2mg/ml); Respigard II	54% / 0 / 23%<br>AEs: Cough (54%), dysgeusia (51%), nausea (37%)	5% vs 12% of possible/probable/proven IA (NS)
Dubois 1995 (31)	Prospective non comparative, N=18	SCT 89%, leukemia induction chemotherapy 11%	30mg/d, 10mg/mL, Respigard II	33% / NR / 22%<br>AEs: dyspnea 33%, vomiting 22%, cough 17%, decrease of peakflow >20% in 50%	NR
De Laurenzi 1996 (32)	Retrospective, historical control<br>N=48 (AmBd) vs historical control (N=107 and N=228 with combination of oral AmB and another systemic antifungal agent as prophylaxis)	SCT 81%	Oral AmB + IV AmB + nebulized AmBd 10 mg/d, 1 mg/ml, with "ACORN" II nebulizer system with adult aerosol MASK (MARQUEST Medical Products - USA)	Good tolerance	0 IFD vs 12.1% in the group oral AmB + azole and 1.6% in the group oral AmB + nystatin (no statistical comparison)
Erjavek 1997 (33)	Prospective non comparative, n= 42	52% AML, 41% ALL,	10mg tid (5 mg/ml), PariInhalerBoy (Pari-	54.8% / 35.7 / 11.9% (21.4% dose reduction)	28% of probable/proven IFD

		7% other haematological diseases	Werk GmbH, Germany)	AEs: coughing, signs of bronchial obstruction, and nausea or vomiting<br>Note: Higher concentration than other studies (5 mg/ml)	
Schwartz 1999 (34)	Randomized, multicenter; n=227 AmBd vs 155 without prophylaxis	74% AML,<br>9% ALL/NHL relapse,<br>17% autoSCT	10mg bid	NR	4.4% vs 5.0% of possible/probable/proven IA (NS)
Morello 2011 (35)	Prospective, non comparative N=102	alloSCT	AmBd 15mg bid (3mg/ml) + systemic antifungal agent (fluconazole 72.5%) ; Airlife Misty-Neb Nebulizer with Aerosol Mask and 213 cm Oxygen Tubing; Cardinal Health Inc., McGaw Park, IL, USA)	89% / 1% / 1% (bronchospasm)<br>AEs: dysgueusia 89%, mild cough 85%, nausea No renal failure	5.0% of possible/probable/proven IFD at day 120. 3/16 in the group of inadequate AmBd (<7days) vs 2/85 in the group of adequate AmBd* (>7days of treatment)
Nihtinen 2012 (36)	Retrospective, historical control N=354 (AmBd) vs 257 (no antifungal prophylaxis)	alloSCT	AmBd 25mg/d for 2-3 months, 5mg/ml, with preadministration of salbutamol	No discontinuation due to treatment	2.5% vs 6.6%* of probable or proven IA over a 5-years period

*: significantly different with $p<0.05$, NR: not reported.

Table S3. Nebulized lipid formulations of AmB (L-AmB or ABLC) as prophylaxis hematological patients.

Ref	Study design	Comorbidities	Dose, Nebulizer	Adverse events / severe AE / treatment–limiting AE / type of AE	Outcome
Alexander 2006 (37)	Prospective, open-label, non comparative, N=40	100% alloSCT	ABLC 50 mg/d d1-4, then o.i.w 13w. + FLU p.o./i.v. day 1 to day 100	7.5 % / 0 / 0<br>AEs: cough, dysgeusia, nausea, vomiting; decrease of FEV1>20% in 5.2% ; no wheezing nor dyspnea	7.5% probable/proven IFD during the study period (13w)
Rijnders 2008 (38)	Randomised, double blinded, versus placebo N= 139 (L-AmB) vs 132 (placebo)	49%AML/MDS, 21% autoSCT, 11% alloSCT	L-AmB 12.5 mg ×2/w until PNN>300 + FLU (dose NR) placebo inh. + FLU (dose NR)<br>adaptive aerosol-delivery system (Halolite or ProDose AAD; Romedic/Medic-Aid; Meerssen, The Netherlands) MMAD=1.9uM	11.5% vs 0.8%* / 0 / 45% vs 30%*<br>AEs: Cough (11.5% vs 0.8%*)	4.3% vs 13.6% of proven/probable IPA*
Slobbe 2008 (39)	Randomised, double blinded, versus placebo N=38 (L-AmB) vs 39 (placebo)	AML-SMD 35%, SCT 48%, Intensive chemotherapy 52%	L-AmB 12.5mg/d ×2/w (5mg/ml)<br>adaptive aerosol-delivery system (Halolite or	32% vs 21% of decline of FEV1>20% (NS)<br>AEs: cough (74% vs 26%*), dysgueusia 37% vs 62% *, nausea or vomiting 13% vs 25%.<br>No creatinine elevation.	NR

			ProDose AAD; Romedic/Medic-Aid; Meerssen, The Netherlands)		
Hullard-Pulstinger 2011 (40)	Prospective, comparative N=96 (L-AmB) vs 105 (Historical control)	67% AML, MDS, AL, 13% ALL, 11% NHL/HD/MM, 7% MPD, 1% AA, PNH, 34% alloSCT	L-AmB 12.5mg + FLU 400 mg vs FLU 400 mg. C= undiluted; 12.5mg/d 4 days then ×2/week, until PNN>500; stream nebulizers (LC Star, Pari, Starnberg, Germany)	36.5% / 0 / 42.7% stopped treatment mainly due to incomfort AEs: Cough > incomfort > bad taste > nausea/vomiting	2.1% vs 3.8% of probable/proven IPA (NS)
Chong 2015 (41)	Prospective, comparative N= 127 vs 108 (Historical control)	86% AML 13% MDS < 1% CML	L-AmB 12.5 mg ×2/w until PNN>500 + FLU 400 mg po vs FLU 400mg Prodose® AAD nebuliser from 2008 to 2011; Akita® AAD nebuliser from 2011; Romedic, Meerssen, The Netherlands)	No serious AEs.	9.5% vs 23.4% of probable/proven IPA* until 28 days after neutrophil recovery

*: significantly different with p<0.05, NR: not reported.

Table S4. Nebulized AmBd as prophylaxis in lung transplant recipients.

Ref	Study design	Comorbidities	Dose, Nebulizer	Adverse events / severe AE / treatment–limiting AE / type of AE	Outcome
Reichenspurner 1997 (42)	Prospective, historical control. N=126 vs 101 without prophylaxis	Hearttransplant 60%, lung transplant 17%, heart-lung transplant 21%	5mg t.i.d increased to 20mg t.i.d in 5 days postop	7.9% (nausea) / 0 / 1.6%	4% vs 7% of fungal infections* (1 year post-op) 2% vs 12% of *Aspergillus* infections
Calvo 1999 (43)	Prospective historical control. N = 52 vs 13 without prophylaxis	Lung transplanted recipients	AmBd 0.2 mg/kg every 8 hours + FLU 200 mg PO BID	NR	O% vs 23% of IFD
Monforte 2001 (44)	Prospetive, historical control N=44 vs 11 without prophylaxis	Lung transplanted recipients	6mgq8h from d1 to d120 postop, then 6mg o.i.d. 1 mg/mL. Jet nebulizer with CR60 compressor	32% / NR / 2.3% (bronchospasm) AEs: Cough, 32%; mild bronchospasm, 9%; nausea, 7%	22.7% vs 72.7% of *Aspergillus* infection (TBA or IPA) (OR=0.13* in multivariate analysis)
Minari 2002 (45)	Retrospective with historical control. N= 81 vs 88 without prophylais	Lung transplanted recipients	AmBd 5-10 mg BID in the immediate postop phase, then switched to itraconazole PO. Up to 2 weeks of overlap	NR	4.9% (Ambd) vs 18.2% (no AmBd) of probable/proven IPA*
Cadena 2009 (46)	Retrospective N=35 (n-AmBd + voriconazole) vs N=32 historical control (itraconazole)	Lung transplanted recipients	AmBd 10 mg BID for 2w + voriconazole 200 mg PO BID for 3 months; Itraconazole 200 mg PO bid for 3months	34% hepatotoxicity (voriconazole) vs 0% (itraconazole)*	2.9% definite IFD vs 12.5% (no stat)

Drew 2004 (47)	Randomised, double blidned, single center N= 49 (AmBd) vs 51 (ABLC)	Lung transplanted recipients	AmBd 25-50 mg daily × 4 d, then weekly for 7w; ABLC 50-100 mg daily × 4 d, then weekly for 7w. Both 5mg/ml,<br><br>Hudson RCI Up-Draft, Model No 1724	AmBd vs ABLC : % of AEs: 42% vs 28% (OR=2.16*)<br><br>AEs: cough (10.6% vs 2.1%); dyspnea (19.9% vs 2.1%); nausea (8.5% vs 2.1%); wheezing 6.4% vs 4.2%; dysgeusia 10.6% vs 7.7%; decline in FEV>20%: 10.6% vs 11.1%; bronchospasm 25% vs 20.4% Discontinuation due to intolerance: 12.2% vs 5.9%	14.3% vs 11.8% of possible/probable/proven IFD during the 2 months of drug administration (NS)
Lowry 2007 (48)	Retrospective, comparative N=27 (AmBd) vs N = 18 (L-AmB)	Lung transplanted recipients	AmBd 10 mg BID L-AMB 20 mg BID	30% vs 30% (NS) (Ratio complaints/doses administered = 1.0% vs 1.2%) / 7% vs 6% (significant respiratory AE requiring treatment) / 0%<br><br>AEs: dyspnea > cough > bronchospasm	5.6% vs 0% of IA (no stat)
Monforte 2010 (49)	Comparative, prospective N=104 vs N=49 (historical control with AmBd)	Lung transplanted recipients	L-AmB 25mg 3×/week × 60 d, then 25 mg 1×/ wk × 120 d, then 25 mg every 2 wk, lifelong; 5mg/ml;<br><br>AmBd 6mg tid (day1-120), then 6mg/d lifelong; 1mg/ml<br><br>1-jet nebulizer (Ventstream® or Sidestream®, Phillips Respironics, Murrysville, PA), CR60 compressor	20.2 vs 24.5% / 0 / 2.9% vs 4.1% (NS) (bronchospasm> nausea)<br><br>AEs (L-AmB vs AmBd): cough 20.2% vs 24.5% (NS); mild dyspnea 7.7% vs 8.2% (NS); nausea 7.7% vs 6.1% (NS) ; bronchospasm 1.9% vs 4.1% (NS)	IA 1.9% vs 4.1% (NS)
Eriksson 2010 (50)	Retrospective, non comparative N=76	Lung transplanted recipients	AmBd 25 mg twice daily (N = 15) or ABLC 50-100 mg daily × 4 d then once weekly from day 5 (N = 61) + Caspo IV in high risk patients and those delayed in inhalation prophylaxis + "occasional" fluconazole 100-200 mg/d during extended ICU stay or use of broad-spectrum antibiotics.	NR	Possible/probable/proven IA 3.9%
Koo 2012 (51)	Retrospective, non comparative N=82 (n-AmBd or n-L-AmB) vs N=83 (n-AmBd or n-L-AmB + micafungine + preemptive strategy)	Lung transplanted recipients	Universal (cohort 1) (N = 82) n-AmBd 25 mg BID OR n-L-AmB 20 mg BID during post-tx hospitalization<br><br>Combination of strategies (cohort 2) (N = 83) universal n-AmBd 25 mg BID or n-L-AmB 20	No AE noted	35.4% probable/proven IFD vs 12% (no statistical analysis)<br>9.8% probable/proven IA vs 2.4%

			mg BID during post-tx hospitalization + MICA 100 mg IV daily during anesthesia and 7-10 d post-tx + Preemptive FLU or VORI (doses not specified) × 3-6 months		
Alissa 2021 (52)	Retrospective N=84 twice daily compared with 3 times daily	Lung transplant recipients	AmBd twice daily compared with 3 times daily	side effects were not significantly higher in the 3 times daily	Rate of Aspergillus infection 17% twice daily vs 4% three times daily twice daily

*: significantly different with p<0.05, NR: not reported.

Table S5. Nebulized AmB lipid formulations as prophylaxis in lung transplant recipients.

Ref	Study design	Comorbidities	Dose, Nebulizer	Adverse events / severe AE / treatment–limiting AE / type of AE	Outcome
Drew 2004 (25)	Rd, db, single center N= 49 AmBd vs 51 ABLC	Lung transplanted recipients	AmBd 25-50 mg daily × 4 d, then weekly for 7w; ABLC 50-100 mg daily × 4 d, then weekly for 7w. Both 5mg/ml, Hudson RCI Up-Draft, Model No 1724	AmBd vs ABLC : 42% vs 28% of AEs (OR=2.16*) AEs: cough (10.6% vs 2.1%); dyspnea (19.9% vs 2.1%); nausea (8.5% vs 2.1%); wheezing 6.4% vs 4.2%; dysgeusia 10.6% vs 7.7%; decline in FEV>20%: 10.6% vs 11.1% ; bronchospasm 25% vs 20.4% discontinuation due to intolerance: 12.2% vs 5.9%	14.3% vs 1.8% of possible/probable/proven IFI during the 2 months of drug administration (NS)
Lowry 2007 (48)	Retrospective, comparative N=27 (AmBd) vs N = 18 (L- AmB)	Lung transplanted recipients	AmBd 10 mg BID L-AMB 20 mg BID	30% vs 30% (NS) (Ratio complaints/doses administered = 1.0% vs 1.2%) / 7% vs 6% (significant respiratory AE requiring treatment) / 0% AEs: dyspnea > cough > bronchospasm	5.6% vs 0% of IA (no stat)
Eriksson 2010 (50)	Retrospective, non comparative N=76	Lung transplanted recipients	AmBd 25 mg twice daily (N = 15) or ABLC 50-100 mg daily × 4 d then once weekly from day 5 (N = 61) + Caspo IV in high risk patients and those delayed in inhalation prophylaxis + “occasional” fluconazole 100-200 mg/d during extended ICU stay or use of broad-spectrum antibiotics.	NR	Possible/probable/proven IA 3.9%
Koo 2012 (51)	Retrospective, non comparative N=82 (n-AmBd or n-L-AmB) vs N=83 (n-	Lung transplanted recipients	Universal (cohort 1) (N = 82) n-AmBd 25 mg BID OR n-L-AmB 20 mg BID during post-tx hospitalization	No AE noted	35.4% probable/proven IFD vs 12% (no stat) 9.8% probable/proven IA vs 2.4%

	AmBd or n-L- AmB and micafungine and preemptive strategy)		Combination of strategies (cohort 2) (N = 83) universal<br><br>n-AmBd 25 mg BID<br><br>or n-L-AmB 20 mg BID during post-tx hospitalization + MICA 100 mg IV daily during anesthesia and 7-10 d post-tx + Preemptive FLU or VORI (doses not specified) × 3-6 months		
Palmer 2001 (53)	Prospective, non comparative, N=51	Lung (92%) or heart- lung (8%) transplant recipients	ABLC 50mg or 100mg (if ventilated)/d for 4 days, then o.i.w for 2 months 5mg/ml (Hudson RCI, Updraft, model 1724)	1.6% / NR / 2%<br>AEs: nausea or vomiting, dysgueusia. Decrease of FEV1>20% in 5%<br>No bronchospasm nor cough nor dyspnea.	4% of pulmonary fungal infections (proven or probable lung candida infection)<br>8% of extra-pulmonary fungal infection (candida)
Borro 2008 (54)	Retrospective, N=60	Lung transplanted recipients (1 heart-lung)	n-ABLC 50 mg (5mg/ml) once every 2 days for 2 wk, then once weekly for 13 wk and FLU 200 mg IV, then 200 mg PO BID for 3 wk Nebulizer (MMAD 3.0 to 4.5uM) with CR60 compressor	6.8% / 0 / 0<br>AE: Nausea, vomitting. No bronchospasm	1.7% possible IPA
Monforte 2010 (49)	Comparative, prospective N=104 vs N=49 (historical control with AmBd)		L-AmB 25mg 3×/week × 60 d, then 25 mg 1×/ wk × 120 d, then 25 mg every 2 wk, lifelong; 5mg/ml;<br><br>AmBd 6mg t.i.d (day1-120), then 6mg/d lifelong; 1mg/ml<br><br>1-jet nebulizer (Ventstream® or Sidestream®, Phillips Respironics, Murrysville, PA), CR60 compressor	20.2 vs 24.5% / 0 / 2.9% vs 4.1% (NS) (bronchospasm> nausea)<br><br>AEs (L-AmB vs AmBd): cough 20.2% vs 24.5% (NS); mild dyspnea 7.7% vs 8.2% (NS); nausea 7.7% vs 6.1% (NS); bronchospasm 1.9% vs 4.1% (NS)	IA 1.9% vs 4.1% (NS)
Monforte 2009 (55)	Prospective, non comparative N=27	Lung transplanted recipients	L-AmB 25 mg (6 ml) 3 times per week up to Day 60 post-transplantation, 25 mg once per week between Days 60 and 180, and 25 mg once every 2 weeks thereafter for life	no changes in the mean FEV1 value before and after n-LAB in 95%. 1 patient had a decrease of 14% (asymptomatic)	Safety and PK study
Peghin 2016 (20)	Retrospective observational N=412	Lung transplanted recipients	L-AmB 25mg ×3/w day 1-60, then 25mg 1x/w day 60-180, then 25mg every 2w. lifetime	(especially hepatotoxicity)	I.A 5.3% (3.6% 1-year cumulative incidence of IA)

Linder 2021 (56)	Retrospective N=105	Lung transplanted recipients	Universal antifungal prophylaxis (ITR 200 mg PO daily 6 months + n-AmBL 12.5 mg three times or ITR) vs (VOR or FLU or MIC)		Universal antifungal prophylaxis more effective than targeted antifungal prophylaxis
Baker 2020 (57)	Prospective N=815	Lung transplanted recipients	n-AmBC		
Ibáñez-Martínez E 2021 (58)	Retrospective	Lung transplanted recipients	nebulized lipidic amphotericin B prophylaxis		
Samanta (59)	Retrospective N=200	Lung transplanted recipients	ISC + n-AmB vs VOR + n-AmB		

*: significantly different with p<0.05, NR: not reported.

Table S6. Comparative studies of nebulized AmBd prophylaxis and nebulized lipid formulations of AmB prophylaxis.

Ref	Study design	Comorbidities	Dose, Nebulizer	Adverse events / severe AE / treatment–limiting AE / type of AE	Outcome
Drew 2004 (47)	Rd, db, single center N= 49 AmBd vs 51 ABLC	Lung transplanted recipients	AmBd 25-50 mg daily × 4 d, then weekly for 7w; ABLC 50-100 mg daily × 4 d, then weekly for 7w. Both 5mg/ml, Hudson RCI Up-Draft, Model No 1724	AmBd vs ABLC : 42% vs 28% (OR=2.16*) cough (10.6% vs 2.1%); dyspnea (19.9% vs 2.1%); nausea (8.5% vs 2.1%); wheezing 6.4% vs 4.2%; dysgeusia 10.6% vs 7.7%; decline in FEV>20%: 10.6% vs 11.1%; bronchospasm 25% vs 20.4% discontinuation due to intolerance: 12.2% vs 5.9%	14.3% vs 11.8% of possible/probable/proven IFI during the 2 months of drug administration (NS)
Lowry 2007 (48)	Retrospective, comparative N=27 (AmBd) vs N =18 (L-AmB)	Lung transplanted recipients	AmBd 10 mg bid L-AMB 20 mg bid	30% vs 30% (NS) (Ratio complaints/doses administered = 1.0% vs 1.2%) / 7% vs 6% (significant respiratory AE requiring treatment) / 0% Type of AE: dyspnea > cough > bronchospasm	5.6% vs 0% of IA (no statistical analysis)
Monforte 2010 (49)	Comparative, prospective N=104 vs N=49 (historical control with AmBd)	Lung transplanted recipients	L-AmB 25mg 3×/week × 60 d, then 25 mg 1×/ wk × 120 d, then 25 mg every 2 wk, lifelong; 5mg/ml; AmBd 6mg t.i.d (day1-120), then 6mg/d lifelong; 1mg/ml 1-jet nebulizer (Ventstream® or Sidestream®, Phillips Respironics, Murrysville, PA), CR60 compressor	20.2 vs 24.5% / 0 / 2.9% vs 4.1% (NS) (bronchospasm> nausea) AEs (L-AmB vs AmBd)): cough 20.2% vs 24.5% (NS); mild dyspnea 7.7% vs 8.2% (NS); nausea 7.7% vs 6.1% (NS); bronchospasm 1.9% vs 4.1% (NS)	IA 1.9% vs 4.1% (NS)

Table S7. Studies of n-voriconazole used as curative treatment for invasive mold infections.

Ref	N	Comorbidity	Pathogen	Pathology	Dilution	Dose	Nebulizer	Other treatments	Outcome
Thanukrishnan 2019 (60)	1	27y., lung transplant (hyper- IgE syndrome)	*Aspergillus* sp.	IPA	Voriconazole 10mg/ml in sterile water	40mg t.i.d 1w., then b.i.d 1w., then 40mg o.i.d on maintenance therapy	AeroEclipse II nebulizer with 8650D compressor operated at 40 psig	IV L-AmB 5mg/kg/d and caspofungin 50mg/d. (systemic voriconazole induced liver toxicity)	Clinical improvement. Died from chronic lung graft dysfunction 3 years later
Hilberg 2012 (61)	3	(1) 66y., high- dose corticosteroids (2) 61y., bilateral lung transplant (3) 37y. heart and bilateral lung transplant	*Aspergillus* sp. (n=3)	IPA (n=3)	Voriconazole 200mg in 20mL of water	40mg (4mL) t.i.d for 2w., then b.i.d. Duration: 6months (1), maintenance therapy (2),	Jet nebuliser (Sidestream®; Philips Respironics), compressor (Portaneb; Medic-Aid); flow rate 8L/min, during 15min	0 (failure or toxicity of systemic voriconazole)	1: Clinical and radiological cure (1) or improvement (2, 3). Patient (2) died 2 months later of graft dysfunction. Good tolerance
Holle 2014 (62)	1	70y, cystic fibrosis	*Scedosporium apiospermum*	Pulmonary IFD	Voriconazole 6.25mg/ml in sterile water	40mg o.i.d, for 3 months	NR	Systemic voriconazole (8mg/kg/d) and L-AmB (3mg/kg/d)	Clinical cureand radiological improvement. Good tolerance
Taton 2018 (21)	1	60y., bilateral lung transplant	*Microascus* sp.	Bronchial IFD	Voriconazole And ABLC	40mg bid and 100mg oid ; duration: 7w.	NR	Combination of IV voriconazole, caspofungin and terbinafine and interventional bronchoscopic treatment	Clinical and bronchoscopic cure

*: significantly different with p<0.05, NR: not reported.

References

1. Birsan T, Taghavi S, Klepetko W. Treatment of aspergillus-related ulcerative tracheobronchitis in lung transplant recipients. J Heart Lung Transplant Off Publ Int Soc Heart Transplant. 1998 Apr;17(4):437–8.
2. Zhou Q-Y, Yang W-J, Zhao X-Q. Pulmonary aspergillosis treated with inhaled amphotericin B. Int J Infect Dis IJID Off Publ Int Soc Infect Dis. 2017 Jan;54:92–4.
3. Huang H, Li Q, Huang Y, Bai C, Wu N, Wang Q, et al. Pseudomembranous necrotizing tracheobronchial aspergillosis: an analysis of 16 cases. Chin Med J (Engl). 2012 Apr;125(7):1236–41.
4. Wu N, Huang Y, Li Q, Bai C, Huang H-D, Yao X-P. Isolated invasive Aspergillus tracheobronchitis: a clinical study of 19 cases. Clin Microbiol Infect Off Publ Eur Soc Clin Microbiol Infect Dis. 2010 Jun;16(6):689–95.
5. Dal Conte I, Riva G, Obert R, Lucchini A, Bechis G, De Rosa G, et al. Tracheobronchial aspergillosis in a patient with AIDS treated with aerosolized amphotericin B combined with itraconazole. Mycoses. 1996 Oct;39(9–10):371–4.
6. Boots RJ, Paterson DL, Allworth AM, Faoagali JL. Successful treatment of post-influenza pseudomembranous necrotising bronchial aspergillosis with liposomal amphotericin, inhaled amphotericin B, gamma interferon and GM-CSF. Thorax. 1999 Nov;54(11):1047–9.
7. Rodenhuis S, Beaumont F, Kauffman HF, Sluiter HJ. Invasive pulmonary aspergillosis in a non-immunosuppressed patient: successful management with systemic amphotericin and flucytosine and inhaled amphotericin. Thorax. 1984 Jan;39(1):78–9.
8. Boettcher H, Bewig B, Hirt SW, Möller F, Cremer J. Topical amphotericin B application in severe bronchial aspergillosis after lung transplantation: report of experiences in 3 cases. J Heart Lung Transplant Off Publ Int Soc Heart Transplant. 2000 Dec;19(12):1224–7.
9. Furco A, Mouchet B, Carbonnelle M, Vallerand H. [Pulmonary mucormycosis: benefit of aerosol amphotericin B?]. Rev Mal Respir. 2001 Jun;18(3):309–13.

10. Alfageme I, Reina A, Gallego J, Reyes N, Torres A. Endobronchial instillations of amphotericin B: complementary treatment for pulmonary mucormycosis. J Bronchol Interv Pulmonol. 2009 Jul;16(3):214–5.
11. McGuire FR, Grinnan DC, Robbins M. Mucormycosis of the bronchial anastomosis: a case of successful medical treatment and historic review. J Heart Lung Transplant Off Publ Int Soc Heart Transplant. 2007 Aug;26(8):857–61.
12. Safdar A, O'Brien S, Kouri IF. Efficacy and feasibility of aerosolized amphotericin B lipid complex therapy in caspofungin breakthrough pulmonary zygomycosis. Bone Marrow Transplant. 2004 Sep;34(5):467–8.
13. Godet C, Cateau E, Rammaert B, Grosset M, Le Moal G, Béraud G, et al. Nebulized Liposomal Amphotericin B for Treatment of Pulmonary Infection Caused by Hormographiella aspergillata: Case Report and Literature Review. Mycopathologia. 2017 Aug;182(7–8):709–13.
14. Castagnola E, Moresco L, Cappelli B, Cuzzubbo D, Moroni C, Lanino E, et al. Nebulized liposomal amphotericin B and combined systemic antifungal therapy for the treatment of severe pulmonary aspergillosis after allogeneic hematopoietic stem cell transplant for a fatal mitochondrial disorder. J Chemother Florence Italy. 2007 Jun;19(3):339–42.
15. Canetti D, Cazzadori A, Adami I, Lifrieri F, Cristino S, Concia E. Aerosolized amphotericin B lipid complex and invasive pulmonary aspergillosis: a case report. Infez Med Riv Period Eziologia Epidemiol Diagn Clin E Ter Delle Patol Infett. 2015 Mar;23(1):44–7.
16. Venanzi E, Martín-Dávila P, López J, Maiz L, de la Pedrosa EG-G, Gioia F, et al. Aerosolized Lipid Amphotericin B for Complementary Therapy and/or Secondary Prophylaxis in Patients with Invasive Pulmonary Aspergillosis: A Single-Center Experience. Mycopathologia. 2019 Apr;184(2):239–50.
17. Safdar A, Rodriguez GH. Aerosolized amphotericin B lipid complex as adjunctive treatment for fungal lung infection in patients with cancer-related immunosuppression and recipients of hematopoietic stem cell transplantation. Pharmacotherapy. 2013 Oct;33(10):1035–43.
18. Morales P, Galán G, Sanmartín E, Monte E, Tarrazona V, Santos M. Intrabronchial instillation of amphotericin B lipid complex: a case report. Transplant Proc. 2009 Aug;41(6):2223–4.
19. Al Yazidi LS, Huynh J, Britton PN, Morrissey CO, Lai T, Westall GP, et al. Endobronchial fusariosis in a child following bilateral lung transplant. Med Mycol Case Rep. 2019 Jan 14;23:77–80.
20. Peghin M, Monforte V, Martin-Gomez M-T, Ruiz-Camps I, Berastegui C, Saez B, et al. 10 years of prophylaxis with nebulized liposomal amphotericin B and the changing epidemiology of Aspergillus spp. infection in lung transplantation. Transpl Int Off J Eur Soc Organ Transplant. 2016 Jan;29(1):51–62.
21. Taton O, Bernier B, Etienne I, Bondue B, Lecomte S, Knoop C, et al. Necrotizing Microascus tracheobronchitis in a bilateral lung transplant recipient. Transpl Infect Dis Off J Transplant Soc. 2018 Feb;20(1).
22. Trujillo H, Fernández-Ruiz M, Gutiérrez E, Sevillano Á, Caravaca-Fontán F, Morales E, et al. Invasive pulmonary aspergillosis associated with COVID-19 in a kidney transplant recipient. Transpl Infect Dis Off J Transplant Soc. 2021 Apr;23(2):e13501.
23. Conneally E, Cafferkey MT, Daly PA, Keane CT, McCann SR. Nebulized amphotericin B as prophylaxis against invasive aspergillosis in granulocytopenic patients. Bone Marrow Transplant. 1990 Jun;5(6):403–6.
24. Jeffery GM, Beard ME, Ikram RB, Chua J, Allen JR, Heaton DC, et al. Intranasal amphotericin B reduces the frequency of invasive aspergillosis in neutropenic patients. Am J Med. 1991 Jun;90(6):685–92.
25. Myers SE, Devine SM, Topper RL, Ondrey M, Chandler C, O'Toole K, et al. A pilot study of prophylactic aerosolized amphotericin B in patients at risk for prolonged neutropenia. Leuk Lymphoma. 1992 Oct;8(3):229–33.
26. Gryn J, Goldberg J, Johnson E, Siegel J, Inzerillo J. The toxicity of daily inhaled amphotericin B. Am J Clin Oncol. 1993 Feb;16(1):43–6.
27. Beyer J, Barzen G, Risse G, Weyer C, Miksits K, Dullenkopf K, et al. Aerosol amphotericin B for prevention of invasive pulmonary aspergillosis. Antimicrob Agents Chemother. 1993 Jun;37(6):1367–9.
28. Heinemann V, Scholz P, Vehling U, Wachholz K, Jehn U. Inhalation of Amphotericin B for Prophylaxis of Invasive Fungal Infections During Intensive Leukemia Therapy. In: Büchner T, Hiddemann W, Wörmann B, Schellong G, Ritter J, editors. Acute Leukemias IV [Internet]. Berlin, Heidelberg: Springer Berlin Heidelberg; 1994 [cited 2019 Mar 7]. p. 762–4. Available from: http://www.springerlink.com/index/10.1007/978-3-642-78350-0_138
29. Hertenstein B, Kern WV, Schmeiser T, Stefanic M, Bunjes D, Wiesneth M, et al. Low incidence of invasive fungal infections after bone marrow transplantation in patients receiving amphotericin B inhalations during neutropenia. Ann Hematol. 1994 Jan;68(1):21–6.
30. Behre GF, Schwartz S, Lenz K, Ludwig W-D, Wandt H, Schilling E, et al. Aerosol amphotericin B inhalations for prevention of invasive pulmonary aspergillosis in neutropenic cancer patients. Ann Hematol. 1995 Dec;71(6):287–91.
31. Dubois J, Bartter T, Gryn J, Pratter MR. The physiologic effects of inhaled amphotericin B. Chest. 1995 Sep;108(3):750–3.
32. De Laurenzi A, Matteocci A, Lanti A, Pescador L, Blandino F, Papetti C. Amphotericin B prophylaxis against invasive fungal infections in neutropenic patients: a single center experience from 1980 to 1995. Infection. 1996 Oct;24(5):361–6.
33. Erjavec Z, Woolthuis GM, de Vries-Hospers HG, Sluiter WJ, Daenen SM, de Pauw B, et al. Tolerance and efficacy of Amphotericin B inhalations for prevention of invasive pulmonary aspergillosis in haematological patients. Eur J Clin Microbiol Infect Dis Off Publ Eur Soc Clin Microbiol. 1997 May;16(5):364–8.
34. Schwartz S, Behre G, Heinemann V, Wandt H, Schilling E, Arning M, et al. Aerosolized amphotericin B inhalations as prophylaxis of invasive aspergillus infections during prolonged neutropenia: results of a prospective randomized multicenter trial. Blood. 1999 Jun 1;93(11):3654–61.

35. Morello E, Pagani L, Coser P, Cavattoni I, Cortelazzo S, Casini M, et al. Addition of aerosolized deoxycholate amphotericin B to systemic prophylaxis to prevent airways invasive fungal infections in allogeneic hematopoietic SCT: a single-center retrospective study. Bone Marrow Transplant. 2011 Jan;46(1):132–6.
36. Nihtinen A, Anttila V-J, Ruutu T, Juvonen E, Volin L. Low incidence of invasive aspergillosis in allogeneic stem cell transplant recipients receiving amphotericin B inhalation prophylaxis. Transpl Infect Dis Off J Transplant Soc. 2012 Feb;14(1):24–32.
37. Alexander BD, Dodds Ashley ES, Addison RM, Alspaugh JA, Chao NJ, Perfect JR. Non-comparative evaluation of the safety of aerosolized amphotericin B lipid complex in patients undergoing allogeneic hematopoietic stem cell transplantation. Transpl Infect Dis Off J Transplant Soc. 2006 Mar;8(1):13–20.
38. Rijnders BJ, Cornelissen JJ, Slobbe L, Becker MJ, Doorduijn JK, Hop WCJ, et al. Aerosolized Liposomal Amphotericin B for the Prevention of Invasive Pulmonary Aspergillosis during Prolonged Neutropenia: A Randomized, Placebo-Controlled Trial. Clin Infect Dis. 2008 May;46(9):1401–8.
39. Slobbe L, Boersma E, Rijnders BJA. Tolerability of prophylactic aerosolized liposomal amphotericin-B and impact on pulmonary function: Data from a randomized placebo-controlled trial. Pulm Pharmacol Ther. 2008 Dec;21(6):855–9.
40. Hullard-Pulstinger A, Holler E, Hahn J, Andreesen R, Krause SW. Prophylactic application of nebulized liposomal amphotericin B in hematologic patients with neutropenia. Onkologie. 2011;34(5):254–8.
41. Chong G-LM, Broekman F, Polinder S, Doorduijn JK, Lugtenburg PJ, Verbon A, et al. Aerosolised liposomal amphotericin B to prevent aspergillosis in acute myeloid leukaemia: Efficacy and cost effectiveness in real-life. Int J Antimicrob Agents. 2015 Jul;46(1):82–7.
42. Reichenspurner H, Gamberg P, Nitschke M, Valantine H, Hunt S, Oyer PE, et al. Significant reduction in the number of fungal infections after lung-, heart-lung, and heart transplantation using aerosolized amphotericin B prophylaxis. Transplant Proc. 1997 Mar;29(1–2):627–8.
43. Calvo V, Borro JM, Morales P, Morcillo A, Vicente R, Tarrazona V, et al. Antifungal prophylaxis during the early postoperative period of lung transplantation. Valencia Lung Transplant Group. Chest. 1999 May;115(5):1301–4.
44. Monforte V, Roman A, Gavalda J, Bravo C, Tenorio L, Ferrer A, et al. Nebulized amphotericin B prophylaxis for Aspergillus infection in lung transplantation: study of risk factors. J Heart Lung Transplant Off Publ Int Soc Heart Transplant. 2001 Dec;20(12):1274–81.
45. Minari A, Husni R, Avery RK, Longworth DL, DeCamp M, Bertin M, et al. The incidence of invasive aspergillosis among solid organ transplant recipients and implications for prophylaxis in lung transplants. Transpl Infect Dis Off J Transplant Soc. 2002 Dec;4(4):195–200.
46. Cadena J, Levine DJ, Angel LF, Maxwell PR, Brady R, Sanchez JF, et al. Antifungal prophylaxis with voriconazole or itraconazole in lung transplant recipients: hepatotoxicity and effectiveness. Am J Transplant Off J Am Soc Transplant Am Soc Transpl Surg. 2009 Sep;9(9):2085–91.
47. Drew RH, Dodds Ashley E, Benjamin DK, Duane Davis R, Palmer SM, Perfect JR. Comparative safety of amphotericin B lipid complex and amphotericin B deoxycholate as aerosolized antifungal prophylaxis in lung-transplant recipients. Transplantation. 2004 Jan 27;77(2):232–7.
48. Lowry CM, Marty FM, Vargas SO, Lee JT, Fiumara K, Deykin A, et al. Safety of aerosolized liposomal versus deoxycholate amphotericin B formulations for prevention of invasive fungal infections following lung transplantation: a retrospective study. Transpl Infect Dis Off J Transplant Soc. 2007 Jun;9(2):121–5.
49. Monforte V, Ussetti P, Gavaldà J, Bravo C, Laporta R, Len O, et al. Feasibility, tolerability, and outcomes of nebulized liposomal amphotericin B for Aspergillus infection prevention in lung transplantation. J Heart Lung Transplant. 2010 May;29(5):523–30.
50. Eriksson M, Lemström K, Suojaranta-Ylinen R, Martelius T, Harjula A, Sipponen J, et al. Control of early Aspergillus mortality after lung transplantation: outcome and risk factors. Transplant Proc. 2010 Dec;42(10):4459–64.
51. Koo S, Kubiak DW, Issa NC, Dietzek A, Boukedes S, Camp PC, et al. A targeted peritransplant antifungal strategy for the prevention of invasive fungal disease after lung transplantation: a sequential cohort analysis. Transplantation. 2012 Aug 15;94(3):281–6.
52. Alissa D, AlMaghrabi R, Nizami I, Saleh A, Al Shamrani A, Alangari N, et al. Nebulized Amphotericin B Dosing Regimen for Aspergillus Prevention After Lung Transplant. Exp Clin Transplant Off J Middle East Soc Organ Transplant. 2021 Jan;19(1):58–63.
53. Palmer SM, Drew RH, Whitehouse JD, Tapson VF, Davis RD, McConnell RR, et al. Safety of aerosolized amphotericin B lipid complex in lung transplant recipients. Transplantation. 2001 Aug 15;72(3):545–8.
54. Borro JM, Solé A, de la Torre M, Pastor A, Fernandez R, Saura A, et al. Efficiency and Safety of Inhaled Amphotericin B Lipid Complex (Abelcet) in the Prophylaxis of Invasive Fungal Infections Following Lung Transplantation. Transplant Proc. 2008 Nov;40(9):3090–3.
55. Monforte V, Ussetti P, López R, Gavaldà J, Bravo C, de Pablo A, et al. Nebulized liposomal amphotericin B prophylaxis for Aspergillus infection in lung transplantation: pharmacokinetics and safety. J Heart Lung Transplant Off Publ Int Soc Heart Transplant. 2009 Feb;28(2):170–5.
56. Linder KA, Kauffman CA, Patel TS, Fitzgerald LJ, Richards BJ, Miceli MH. Evaluation of targeted versus universal prophylaxis for the prevention of invasive fungal infections following lung transplantation. Transpl Infect Dis Off J Transplant Soc. 2021 Feb;23(1):e13448.

57. Baker AW, Maziarz EK, Arnold CJ, Johnson MD, Workman AD, Reynolds JM, et al. Invasive Fungal Infection After Lung Transplantation: Epidemiology in the Setting of Antifungal Prophylaxis. Clin Infect Dis Off Publ Infect Dis Soc Am. 2020 Jan 1;70(1):30–9.
58. Ibáñez-Martínez E, Solé A, Cañada-Martínez A, Muñoz-Núñez CF, Pastor A, Montull B, et al. Invasive scedosporiosis in lung transplant recipients: A nine-year retrospective study in a tertiary care hospital. Rev Iberoam Micol. 2021 Dec;38(4):184–7.
59. Samanta P, Clancy CJ, Marini RV, Rivosecchi RM, McCreary EK, Shields RK, et al. Isavuconazole Is as Effective as and Better Tolerated Than Voriconazole for Antifungal Prophylaxis in Lung Transplant Recipients. Clin Infect Dis Off Publ Infect Dis Soc Am. 2021 Aug 2;73(3):416–26.
60. Thanukrishnan H, Corcoran TE, Iasella CJ, Moore CA, Nero JA, Morrell MR, et al. Aerosolization of second-generation triazoles: In vitro evaluation and application in therapy of invasive airway aspergillosis. Transplantation. 2019 Mar 4;
61. Hilberg O, Andersen CU, Henning O, Lundby T, Mortensen J, Bendstrup E. Remarkably efficient inhaled antifungal monotherapy for invasive pulmonary aspergillosis. Eur Respir J. 2012 Jul;40(1):271–3.
62. Holle J, Leichsenring M, Meissner PE. Nebulized voriconazole in infections with Scedosporium apiospermum--case report and review of the literature. J Cyst Fibros Off J Eur Cyst Fibros Soc. 2014 Jul;13(4):400–2.
